# Multifaceted functions of Drp1 in hypoxia/ischemia-induced mitochondrial quality imbalance: from regulatory mechanism to targeted therapeutic strategy

**DOI:** 10.1186/s40779-023-00482-8

**Published:** 2023-10-13

**Authors:** Shuai Hao, He Huang, Rui-Yan Ma, Xue Zeng, Chen-Yang Duan

**Affiliations:** 1 Department of Anesthesiology, the Second Affiliated Hospital of Chongqing Medical University, Chongqing, 400010 China; 2 Research Institute of General Surgery, Jinling Hospital, Affiliated Hospital of Medical School, Nanjing University, Nanjing, 210002 China; 3 Department of Cardiovascular Surgery, Xinqiao Hospital, Army Medical University, Chongqing, 400037 China; 4 Institute for Brain Science and Disease, Chongqing Medical University, Chongqing, 400010 China

**Keywords:** Dynamin-related protein 1 (Drp1), Hypoxic-ischemic injury, Mitochondrial quality imbalance, Cell dysfunction, Organ damage

## Abstract

Hypoxic-ischemic injury is a common pathological dysfunction in clinical settings. Mitochondria are sensitive organelles that are readily damaged following ischemia and hypoxia. Dynamin-related protein 1 (Drp1) regulates mitochondrial quality and cellular functions via its oligomeric changes and multiple modifications, which plays a role in mediating the induction of multiple organ damage during hypoxic-ischemic injury. However, there is active controversy and gaps in knowledge regarding the modification, protein interaction, and functions of Drp1, which both hinder and promote development of Drp1 as a novel therapeutic target. Here, we summarize recent findings on the oligomeric changes, modification types, and protein interactions of Drp1 in various hypoxic-ischemic diseases, as well as the Drp1-mediated regulation of mitochondrial quality and cell functions following ischemia and hypoxia. Additionally, potential clinical translation prospects for targeting Drp1 are discussed. This review provides new ideas and targets for proactive interventions on multiple organ damage induced by various hypoxic-ischemic diseases.

## Background

Hypoxic-ischemic injury is a common, irreversible condition for which effective treatment is currently lacking. Ischemia and hypoxia can result from failure of the respiratory system, insufficient blood perfusion of vital organs, and dysfunctional or insufficient hemoglobin. Hemorrhagic shock, ischemic stroke, heart failure, and coronary heart disease are examples of prevalent hypoxic-ischemic diseases [1]. A long-term hypoxic microenvironment is commonly observed with aging and chronic diseases, such as tumors and diabetes [2]. In the recent pandemic of corona virus disease 2019 (COVID-19), patients infected with the causal virus severe acute respiratory syndrome coronavirus 2 (SARS-CoV-2) often presented with life-threatening hypoxemia without dyspnea or signs of respiratory distress, a condition termed “silent hypoxia” [3] that has been associated with an increased mortality risk and poor survival [4]. In refractory vasodilatory septic shock, mechanical circulatory support cannot improve clinical outcomes and may worsen hemodynamics [5]. Restoring blood pressure and improving tissue gas exchange alone cannot improve the survival rate after resuscitation in intensive care unit patients [6]. These contradictions have challenged our understanding of the pathological mechanisms of ischemia/hypoxia-induced multiple organ dysfunction (MOD).

Oxygen deprivation during ischemia/hypoxia decreases adenosine triphosphate (ATP) production and brings subsequent changes in ion influx, acidosis, and cell swelling, causing cell death and necrosis [7]. Mitochondria are among the first organelles that are damaged during ischemia and hypoxia [8], constituting the first line of defense against inflammation and viruses, such as SARS-CoV-2 [9]. There has been significant progress in understanding the close relationship between mitochondrial quality imbalance and MOD after hypoxic-ischemic injury [10], with extensive focus on the critical role of mitochondrial dynamin-related protein 1 (Drp1) in this context [11]. Drp1 is a highly conserved protein found in eukaryotic cells, which serves as a critical mediator of mitochondrial fission. Typically, Drp1 exists in the cytoplasm and translocates to the mitochondrial outer membrane (MOM), where it assembles into a spiral-like structure exerting a mechanical force on the mitochondria, leading to constriction and subsequent fission [12]. However, the significance of Drp1 conformational changes and modifications in different hypoxic-ischemic injury models remains controversial, possibly due to limited insights into the regulation of Drp1 under conditions of ischemia and hypoxia.

To help resolve these gaps in knowledge, in this study, we summarize the updated research progress on the role and regulatory mechanisms of Drp1 oligomeric changes, modification types, and protein interactions in different types of hypoxic-ischemic injury, focusing on a systematic review of our previous work [11, 13–16] and other high-quality studies. Controversial topics and discrepant conclusions put forth in the literature are also addressed. Furthermore, we provide future directions for studying Drp1-mediated mitochondrial quality imbalance and its potential role in the protection against hypoxic-ischemic injury as an emerging therapeutic target.

## Oligomeric changes of Drp1: structural basis for its functionality in ischemia and hypoxia

Drp1 is a mechanochemical protein encoded by the *DNM1L* gene and is a member of the dynamin-like GTPase superfamily. Although the functional domains of Drp1 have been briefly characterized [17], their functional significance has not been thoroughly explored. Moreover, there have been no comprehensive reviews on the oligomeric changes of Drp1 and their relationship with protein localization and functions. Uncovering these relationships is essential for gaining a better understanding of the biological significance of Drp1 in ischemia and hypoxia.

### Functional significance of Drp1 domains

**Fig. 1:**
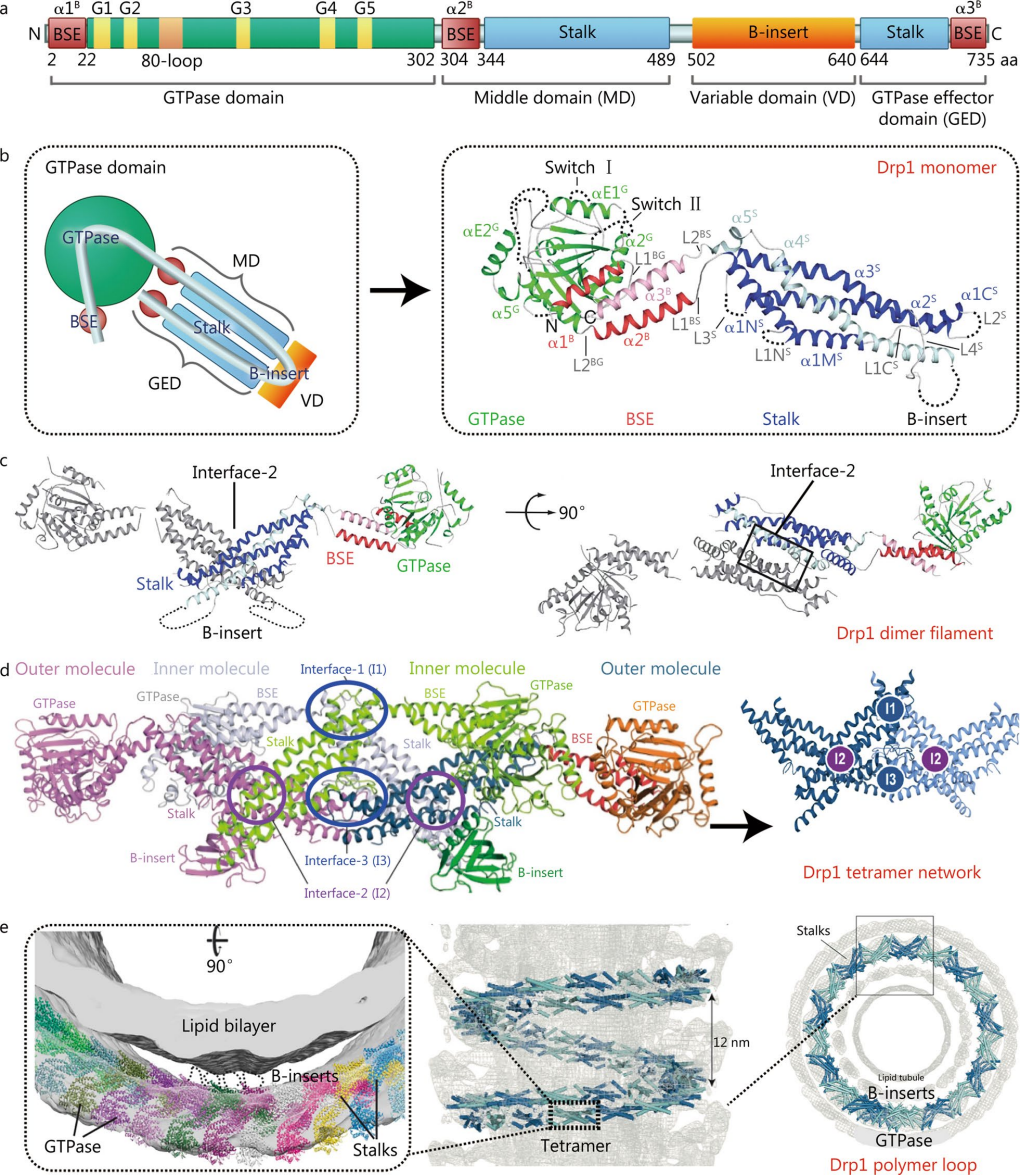
Drp1 oligomerization-related changes. **a** Secondary structure of the Drp1 peptide chain and its functional domains. The five motifs in the GTPase domain are essential features of Drp1, which is a large GTPase protein in the dynamin superfamily. These features include the G1 motif (32–39 aa), G2 motif (58–60 aa), 80-loop (72–87 aa), G3 motif (146–149 aa), G4 motif (215–218 aa), and G5 motif (245–248 aa). The 80-loop is a unique structure that distinguishes Drp1 from other dynamin superfamily members. **b** Tertiary structure of the Drp1 monomer. The bundle signaling element (BSE) conformation comprises three helix bundles (α1^B^, α2^B^, and α3^B^), which mainly regulate the nucleotide-dependent oligomeric changes from the GTPase domain to the Stalk region. The Stalk conformation comprises elongated, antiparallel four-helix bundles; the first three belong to the MD domain (α1^S^, α2^S^, and α3^S^). The α1^S^ helix is further subdivided into α1N^S^, α1M^S^, and α1C^S^, which are connected by two loops (L1N^S^ and L1C^S^). α4^S^ belongs to the GTPase effector domain (GED) domain and connects with α3^S^ via L4.^S^ after crossing the B-insert domain. **c** Quaternary conformation of the Drp1 dimer filament. Side and top views of the Drp1 dimer that assembles via the central Stalk interface-2. **d** Quaternary conformation of the Drp1 tetramer network. All GTPase domains locate on the same side, and all B-insert domains locate on the opposite side in the Drp1 tetramer conformation. **e** Quaternary conformation of the Drp1 polymer loop. All B-insert regions locate inside the Drp1 polymer loop, which is responsible for recognizing and interacting with mitochondrial membrane proteins. Drp1 Dynamin-related protein 1

The Drp1 protein consists of four key functional domains: the N-terminal GTPase domain (2 – 302 aa), middle domain (MD, 304 – 489 aa), variable domain (VD, 502 – 640 aa), and C-terminal GTPase effector domain (GED, 644 – 735 aa) [18] (Fig. 1a). The GTPase domain is responsible for binding and hydrolyzing guanosine triphosphate (GTP) and can be further divided into five motifs: G1, G2, G3, G4, and G5. The G1 motif binds to the phosphate anion of GTP [19], the G2 and G3 motifs are involved in Mg^2+^ binding and GTP hydrolysis [20], G4 binds to GTP [21], and G5 binds to ribosomes [19]. Continuous GTP binding and hydrolysis regulated by these five motifs constitute the switch for oligomeric changes of Drp1 [22, 23], with mutations in the GTPase domain potentially having variable effects on its enzymatic activity under ischemia and hypoxia [24].

The MD and GED are largely composed of Stalk regions [25–27], whereas the VD comprises the B-insert region. The bundle signaling element (BSE) regions are located at the N-(α1^B^) and C-(α2^B^) termini of the GTPase domain and at the C-terminus of the GED (α3^B^) [28]. The GTPase-BSE-Stalk-B-insert tertiary structure folded by the Drp1 peptide chain is the basis for oligomeric changes, with the BSE and Stalk regions acting as binding sites [18], while the B-insert region is mainly subjected to post-translational modifications during ischemia and hypoxia [29] (Fig. 1b).

### Biological significance of Drp1 oligomeric changes

Drp1 has three oligomeric forms: a dimer (Drp1 filament), tetramer (Drp1 network), and polymer (Drp1 loop). Oligomerization of Drp1 occurs progressively. First, two Drp1 monomers interact at the central Stalk regions (interface-2) to form the Drp1 dimer [26, 30, 31] (Fig. 1c). Next, two Drp1 dimers interact at the peripheral Stalk regions (interface-1 and -3) to form the Drp1 tetramer network with four interacting Stalk regions [30] (Fig. 1d). Finally, approximately 48 Drp1 tetramers bend and join together to form a double-helix tube, known as the Drp1 polymer loop, which wraps around the MOM [30] (Fig. 1e).

Under normal circumstances, cytoplasmic Drp1 (Cyto-Drp1) maintains the oligomeric balance of dimers and tetramers, while mitochondrial Drp1 (Mito-Drp1) mainly presents as a polymer loop [32]. However, ischemia and hypoxia can disrupt Drp1 homeostasis by interfering with binding in the Stalk regions [33], thus causing abnormal mitochondrial fission, such as excessive fission or pathological non-fission. Thus, Drp1 oligomeric changes are the structural basis for its function as a mechanochemical enzyme in ischemia and hypoxia.

## Post-transcriptional modifications of Drp1

Post-transcriptional modifications of Drp1 are essential for its function in mitochondrial quality control that plays critical roles in ischemia- and hypoxia-related diseases. These modifications mainly occur within the B-insert region of Drp1 and determine this domain’s functional properties in plasma membrane interactions. Known Drp1 modification types include phosphorylation, SUMOylation, ubiquitination, S-nitrosylation, O-GlcNAcylation, and acetylation which may influence Drp1 subcellular localization, protein interactions, and stability, among other aspects, during ischemia and hypoxia.

### Phosphorylation of Drp1

Phosphorylation is the major modification of Drp1, with Ser616 and Ser637 being the two most actively phosphorylated sites. Other phosphorylation sites of functional significance include Ser40, Ser44, Ser585 [34], Ser412, Ser684 [35], Ser579, and Ser600 [36].

#### Phosphorylation of Drp1 at Ser616

Phosphorylation at Ser616 promotes the translocation of Drp1 to the mitochondria and subsequent mitochondrial fission, thus impacting caspase-induced apoptosis [37]. However, phosphorylation does not affect the oligomeric changes and GTPase activity of Drp1 [38].

Extracellular-signal regulated kinase (ERK1/2), PTEN-induced kinase 1 (PINK1), phosphoglycerate mutase 5 (PGAM5), receptor interacting protein 1 (RIP1)/RIP3, protein kinase B (Akt), uncoupling protein-2 (UCP-2), cyclin-dependent kinase 5 (CDK5), CDK1/cyclin B, and LIGHT can all increase Drp1-Ser616 phosphorylation [39, 40], while dual-specificity phosphatase 6 (DUSP6) can decrease phosphorylation [41]. In the myocardial ischemia–reperfusion rat model, PGAM5 upregulation activates the RIP1-RIP3 pathway, resulting in Drp1-Ser616 phosphorylation and subsequent mitochondrial dysfunction. This, in turn, causes decreased mitochondrial membrane potential (MMP) and increased reactive oxygen species (ROS) production, which accelerate myocardial cell necrosis. Moreover, the positive feedback loop between RIP1-RIP3 signaling and Drp1-Ser616 phosphorylation may promote creatine kinase release and exacerbate myocardial injury [42]. In pulmonary hypertension, hypoxia-inducible factor-1α (HIF-1α) upregulation in pulmonary vascular smooth muscle cells can induce Drp1-Ser616 phosphorylation via CDK1/cyclin B, resulting in excessive mitochondrial fission [43]. In the liver ischemia–reperfusion mouse model, the decrease in several microRNAs, including miR-410-3p, miR-490-3p, and miR-582-5p, can upregulate Drp1-Ser616 phosphorylation via CDK1/cyclin B, resulting in the exacerbation of hepatocyte apoptosis [44]. In renal ischemia–reperfusion, activation of LIGHT-HVEM/LTβR signaling upregulates Drp1-Ser616 phosphorylation, leading to excessive fission, reduced mitochondrial DNA (mtDNA) content, and impaired mitophagy, which are associated with high mortality and chronic kidney disease conversion rates in acute kidney injury [45]. During brain injury, Drp1-Ser616 phosphorylation by PINK1 significantly impacts the distribution of mitochondria in neurons, which, in turn, affects neural circuits and synaptic connections [46]. In hypoxemia caused by COVID-19, SARS-CoV-2 activates Drp1 via phosphorylation at Ser616 through the RIP1/RIP3 [47], resulting in alveolar injury and pulmonary vasoconstriction dysfunction [48].

#### Phosphorylation of Drp1 at Ser637

We previously demonstrated that variations in Drp1-Ser637 phosphorylation depend on the duration of hypoxia/ischemia exposure [14]. Short-term severe ischemic/hypoxic stimuli reduce Drp1-Ser637 phosphorylation, while long-term preconditioning may increase Drp1-Ser637 phosphorylation. This is also consistent with the dynamic regulation of mitochondrial quality by Drp1. Furthermore, Drp1-Ser637 phosphorylation follows a periodic pattern, directly affecting the circadian rhythm of mitochondrial ATP production [49].

Calcium-calmodulin kinase (CaMK) IIα and RhoA/Rho-associated kinase 1 (ROCK1) can upregulate Drp1-Ser637 phosphorylation, while glycogen synthase kinase-3β (GSK-3β), calcineurin, protein kinase A (PKA), and PKA inhibitor (PKI) may suppress phosphorylation. In acute hypoxic ischemia, decreased Drp1-Ser637 phosphorylation promotes mitochondrial fission and apoptosis, in addition to inhibiting mitophagy [11]. However, during chronic hypoxic ischemia, Drp1-Ser637 phosphorylation is moderately increased to adapt to the hypoxic environment, promoting mitophagy and preventing apoptosis, thus preserving sub-healthy mitochondria that help survival during long-term hypoxic conditions [50]. In the early stage of cerebral ischemia, increased RIP3 expression leads to RIP1-RIP3 interactions and decreased Drp1-Ser637 phosphorylation, resulting in the mitochondrial translocation of Drp1. However, hypoxia preconditioning can increase Drp1-Ser637 phosphorylation by influencing CaMKIIα activity and its interaction with RIP1, which in turn, reduces mitochondrial fission and apoptosis [51]. In myocardial ischemia–reperfusion, calcineurin increases the mitochondrial matrix calcium level by inhibiting Drp1-Ser637 phosphorylation, resulting in left ventricular dysfunction [52]. Moreover, miR-499 was shown to inhibit cardiomyocyte apoptosis through the suppression of calcineurin-mediated Drp1-Ser637 dephosphorylation [53]. In hepatic ischemia–reperfusion, decreased Drp1-Ser637 phosphorylation caused by calcineurin can accelerate the onset of hepatic encephalopathy [54]. In myocardial infarction, the upregulation of ubiquitin ligase SIAH2 can suppress Drp1-Ser637 phosphorylation via the A-kinase anchoring protein 121 (AKAP121)/PKA pathway, thereby enhancing Drp1-mitochondrial fission protein 1 (Fis1) interactions and accelerating mitochondrial fission and cardiomyocyte apoptosis [55].

### SUMOylation of Drp1

The SUMOylation of Drp1 can affect numerous processes, such as its binding to the outer mitochondrial membrane, mitochondrial contraction induced by contact between the endoplasmic reticulum and mitochondria (ER-Mito contact) [56], ER calcium flux, mitochondrial cristae remodeling, and cytochrome C release [57]. An increase in SUMOylation of Drp1 is observed in a state of acute ischemia and its decrease occurs in a state of chronic hypoxia, which may lead to mitochondrial quality imbalance and vital organ dysfunction. The reported SUMOylation modification sites of Drp1 include K557, K560, K569, and K571, identified by Yamada et al. [58], as well as K532, K535, K558, K568, K594, K597, K606, and K608, identified by Adaniya et al. [59]. However, these require further confirmation.

SUMO-specific proteases (SENPs) can influence Drp1 SUMOylation, thus affecting mitochondrial morphology and ER tubulation. SENP3 and SENP5L reportedly can downregulate Drp1 SUMOylation [60], whereas SENP5S promotes SUMOylation [58]. In hepatic ischemia–reperfusion, the SUMOylation of Drp1 is significantly increased in the transplanted liver, influencing liver regeneration by protecting mitochondrial morphology via downregulation of the SUMO-E1 enzyme UBA2 and Drp1 SUMOylation [61]. In ischemia-hypoxia-induced apoptosis, activation of the SUMO-E3 mitochondrial-anchored protein ligase (MAPL)/mitochondrial ubiquitin ligase 1 (MUL1) induces Drp1 SUMOylation, accelerating the formation of ER-Mito contacts and promoting mitochondrial fission as well as cytochrome C release [56].

### Ubiquitination of Drp1

E3 ubiquitin ligases, such as MARCH5, Parkin, and anaphase-promoting complex/cyclosome and its coactivator cadherin 1 (APC/Cdh1), can ubiquitinate Drp1, causing its degradation. Conversely, ovarian tumor-associated protease deubiquitinase 6A (OTUD6A) de-ubiquitinates Drp1, promoting its stability [62]. The specific sites of Drp1 ubiquitination remain unclear; however, these are speculated to be within the MD or B-insert region.

In hypoxic injury, Parkin-mediated Drp1 ubiquitination initiates mitophagy through a proteasome-dependent pathway and eliminates oxidatively damaged mitochondria [63]. In chronic debilitating neuropathy, MITOL/MARCH5 can interact with mitofusin 2 (MFN2) to induce Drp1 ubiquitination and protein degradation, resulting in mitochondrial hyperfusion [64]. In addition, APC/Cdh1-mediated Drp1 ubiquitination promotes the formation of mitochondrial tubules and reticular structures [65].

### S-nitrosylation of Drp1

The Cys644 site in the Stalk region of Drp1 can be S-nitrosylated, which is essential for Drp1 dimerization and its GTPase activity [66, 67]. However, Drp1 S-nitrosylation does not directly regulate its activity but instead causes mitochondrial fission by increasing Drp1 phosphorylation at Ser616 [68].

In cerebral ischemia–reperfusion, increased ONOO^−^ can induce Drp1 S-nitrosylation, leading to massive Drp1 recruitment to damaged mitochondria, thereby increasing mitophagy and exacerbating brain injury. Inhibiting the formation of 3-nitrotyrosine can reduce Drp1 S-nitrosylation as well as the expression of NADPH oxidase and induced nitric oxide synthase, thus reducing cerebral infarction and improving neurological function [69]. In subarachnoid hemorrhage, increased nitric oxide induces Drp1 S-nitrosylation, increasing mitochondrial fission and dysregulating synaptic plasticity in the cortical and hippocampal neurons [70]. However, S-nitrosoglutathione reductase can attenuate Drp1 S-nitrosylation and reduce mitochondrial fission and neuronal apoptosis, thus exerting a neuroprotective effect [71]. Moreover, Drp1 S-nitrosylation induced by stress increases ER transport of ceramide synthase 1 to the mitochondria via p17, regulating lipid metabolism at the MOM and affecting several processes, such as mitochondrial ceramide production, mitophagy [72], and apoptosis [73]. Additionally, dihydropteridine reductase can affect mitochondrial morphology and cell homeostasis by regulating Drp1 S-nitrosylation [74].

### O-GlcNAcylation of Drp1

The O-GlcNAcylation of Drp1 can upregulate its GTP-activated form and accelerate its translocation from the cytoplasm to mitochondria. The Thr585 and Thr595 sites of Drp1 can undergo O-GlcNAcylation [75], which may lead to decreased Ser637 phosphorylation [76].

In neurodegeneration, β-amyloid can regulate mitochondrial fission by increasing Drp1 O-GlcNAcylation [77]. Additional O-GlcNAcylation modification sites may exist within the GTPase domain of Drp1 [78]. Moreover, we previously found that Drp1—leucine-rich repeat kinase 2 (LRRK2) protein coupling after hemorrhagic shock also occurs at the Drp1 Thr595 site [13]. However, it remains unclear whether this effect is related to changes induced by Drp1 O-GlcNAcylation.

### Acetylation of Drp1

In a study of cardiac insufficiency due to lipid overload, Hu et al. [79] first reported that Drp1 undergoes acetylation, resulting in increased Drp1-Ser616 phosphorylation and Drp1 protein expression. This causes myocardial fibrosis and hypertrophy as well as cardiomyocyte death eventually leading to cardiac dysfunction, suggesting that acetylation of Drp1 may be closely related to dysregulated cellular metabolism. However, the acetylation of Drp1 in hypoxic-ischemic injury requires further investigation.

## Protein interactions of Drp1

As the Drp1 protein lacks a pleckstrin homology (PH) domain to bind membrane phospholipids, its binding to the MOM and peroxisomal membrane requires receptors or interacting proteins, such as mitochondrial fission factor (MFF), mitochondrial dynamics protein-49 (MiD49), MiD51, Fis1, and LRRK2. Interference with the expression of these proteins does not affect Drp1 levels, suggesting that these receptor proteins do not influence Drp1 expression [80]. However, under ischemic-hypoxic conditions, binding of Drp1 to different receptors or interacting proteins affects mitochondrial fission patterns, guiding mitochondrial and cell fates [81].

### MFF-Drp1 interactions

MFF is distributed on both the MOM and peroxisomal membrane [82, 83]. In cardiac tissues, MFF is the predominant Drp1 receptor. The binding site of MFF on the B-insert region of Drp1 is normally obscured in the quaternary structure. Under hypoxic conditions, this domain binds cardiolipin on the MOM, induces the opening of the MFF binding site of Drp1, and reduces the curvature of the mitochondrial constriction site, which, in turn, promotes formation of the Drp1 polymer loop around the MOM [84].

### MiD-Drp1 interactions

MiD49 and MiD51 are distributed on the MOM [85]. By binding to Drp1, MiD49 and MiD51 promote the formation of mitochondrial fission complexes, which are critical for maintaining proper mitochondrial dynamics and cellular homeostasis.

#### MiD49-Drp1 interactions

The binding of MiD49 to Drp1 is regulated by GTP-dependent oligomeric changes of Drp1. In ischemia and hypoxia, the interaction between MiD49 and Drp1 is a “binding into a loop and then dissociating” process.

Drp1 has several binding sites for MiD49. The GTPase domain of Drp1 can bind to the dynamic-related region (DRR) domain of MiD49, which is vital for the gradual bending of Drp1 dimer filaments into a polymer loop around the MOM. Typically, the binding sites for MiD49 are covered in Drp1 dimer filaments. However, after ischemia and hypoxia, the binding sites are exposed through the action of GTP. With prolonged GTP-dependent activity, MiD49 can bind the L1N^S^ loop in the Stalk region and the Ser637 site of Drp1, whereas phosphorylation of Drp1-Ser637 inhibits the Drp1-MiD49 interaction [86].

When all of the GTP is hydrolyzed to GDP, Drp1 dissociates from MiD49, leaving the Drp1 loop behind. When GTP is exhausted and MiD49 is completely dissociated from Drp1, the loop plays further roles in mitochondrial contraction and fission [86]. This may explain why exogenous MiD49 supplementation inhibits mitochondrial fission in ischemia and hypoxia [87].

#### MiD51-Drp1 interactions

MiD51 has a unique adenosine diphosphate (ADP)-binding capacity [85]. In the absence of ADP, exogenous MiD51 inhibits Drp1-mediated mitochondrial fission. In contrast, with sufficient ADP, exogenous MiD51 promotes Drp1-mediated mitochondrial fission [87]. Drp1 binding to MiD51 is influenced by multiple factors, such as the GTP binding and polymerization of Drp1 as well as MiD51 dimerization [88]. In the presence of Mg^2+^, only GTP can enable Drp1 binding to MiD51 [86].

In light of cell type-specific differences, the exact role of the MiD51 receptor in Drp1-mediated mitochondrial fission is still debated [85, 89]. We propose that this complex binding process may be associated with the different states of the specific energy forms [i.e., GTP/guanosine diphosphate (GDP) and ATP/ADP]. As the microenvironment strongly influences MiD-Drp1 interactions, we caution against using exogenous MiD or cellular-level simulation in future studies so as to avoid false-positive results.

#### Complementary effects of MiDs and MFF on Drp1 interactions

Although MiD49, MiD51, and MFF regulate Drp1 recruitment and activity differently, they exhibit complementary effects on Drp1 interactions [90]. MFF selectively recruits activated Drp1 polymers, while MiD49 and MiD51 recruit Drp1 in the GTP-bound state. In pulmonary hypertension, increased MiDs expression in pulmonary vascular smooth muscles can promote Drp1-mediated mitochondrial fission [91], whereas downregulating the expression of MiDs can disrupt the interactions between Drp1 and MFF, thus promoting mitochondrial fusion [92]. In addition, MFF-Drp1 interactions can inhibit the ubiquitin degradation of MiD49 by negatively regulating the activity of the E3 ubiquitin ligase MARCH5 in the outer mitochondrial membrane. Knockdown of *Drp1* or *MFF* increases MiD49 ubiquitination and decreases its expression, whereas further *MARCH5* knockdown blocks the effects of MFF-Drp1 interactions on MiD49 [93]. These findings suggest that the relationship among Drp1, MFF, and MiDs is not limited to ligand-receptor interactions but also involves multiple mutual regulatory effects, the mechanisms of which may be closely related to Drp1 post-transcriptional modifications in ischemia and hypoxia.

### Fis1-Drp1 interactions

Fis1-Drp1 interactions are involved in Drp1-mediated mitochondrial peripheral fission primarily by recruiting lysosomes, a newly discovered fission mode distinct from the mitochondrial midzone fission mediated by MFF- and MiD-Drp1 interactions [81]. In this process, Fis1-Drp1 interactions can regulate the oligomerization of MiD51 through a feedback mechanism [94]. In myocardial ischemia, the ubiquitin ligase SIAH2 can increase the Fis1-Drp1 interactions by inhibiting the PKA pathway, resulting in excessive mitochondrial fission and cardiomyocyte apoptosis [55]. The phosphorylation of Fis1 at the T34 and Y38 sites can trigger the assembly of Mito-Drp1 and promote mitochondrial fission [95, 96]. In myocardial ischemia–reperfusion injury, Fis1 phosphorylation may cause greater damage to mitochondrial quality than Drp1 phosphorylation [97]. However, Fis1 and Drp1 may not bind directly [80] but might instead inhibit mitochondrial fusion by suppressing the GTPase activity of mitochondrial fusion proteins, such as MFN1, MFN2, and optic atrophy 1 (OPA1) [98]. In *Drp1*-knockout cells, the upregulation of Fis1 can also regulate mitochondrial quality control [99]. Therefore, the requirement of Fis1 for Drp1-mediated mitochondrial fission remains debatable.

### LRRK2-Drp1 interactions

Diminished LRRK2 activity causes F-actin hyperstabilization and Drp1 mislocalization [100]. In Parkinson’s disease, LRRK2-Drp1 interactions induce neurotoxicity via the microtubule-binding protein tau [101]. We recently showed that in ischemia and hypoxia, Mito-Drp1 can also bind to the inactivated form of LRRK2, leading to excessive opening of the mitochondrial permeability transition pore (mPTP) by inducing the release of hexokinase 2 from its interaction with the mPTP at the inner mitochondrial membrane (IMM) [13]. *LRRK2*, *R1441C*, and *G2019S* mutations may accelerate this process [102]. Additionally, LRRK2 may indirectly regulate the activity and protein interactions of Drp1 through mitophagy or lysosomes [103]. However, the specific mechanism requires further investigation.

## Regulatory mechanisms of Drp1 in ischemia and hypoxia

Based on oligomerization changes and modifications, Drp1 can have various functions in ischemia and hypoxia via binding various proteins. These functions include direct regulation of mitochondrial quality, such as mitochondrial fission, mitochondrial bioenergetics, and mitophagy, as well as regulating cellular functions, such as cell death, cytoskeleton stability, and vesicle formation and release.

### Functions of Drp1 in mitochondrial fission

Mediating mitochondrial fission is the classical and primary function of Drp1 in ischemia and hypoxia. The traditional understanding of mitochondrial fission process can be summarized as follows. First, the mitochondrial nucleoid formed by replicating mtDNA determines the location of ER recruitment on the MOM [104], inducing actin polymerization and bundling at the ER-Mito contact site [105]. The formation of ER-Mito contacts leads to mitochondrial contraction [106, 107], allowing Mito-Drp1 adaptors (such as MFFs and MiDs) [108] and Cyto-Drp1 to aggregate into a Drp1 polymer loop at the mitochondrial contraction site, a process that is dependent on the GTP binding ability of Drp1 [109]. The Mito-Drp1 polymer loop then further cleaves the phospholipid bilayer of the mitochondrial membrane, a process that is dependent on GTP hydrolysis by Drp1 [86, 110]. Finally, Dynamin-2 is recruited to the Mito-Drp1 polymer loop and assists in completing mitochondrial membrane cleavage, forming two daughter mitochondria [111].

In this traditional understanding of mitochondrion fission, Drp1 is passively recruited to the mitochondria to partially cleave the membrane after ER-Mito contact-induced mitochondrial contraction. However, many studies have confirmed that Drp1 plays a crucial role in several steps of the fission process, not only after the formation of ER-Mito contacts [81, 112]. Moreover, Fonseca et al. [113] revealed that mitochondrial fission and outer membrane cleavage were only affected by Drp1, independent of Dynamin-2 and regardless of physiological or pathological conditions.

In ischemia and hypoxia, Cyto-Drp1 tetramers/dimers can translocate to the lysosomes, ER, and mitochondria through distinct oligomerization changes and modifications, thus acting at different stages throughout the mitochondrial fission process. This involves calcium signaling and vesicle transport between various organelles, including the mitochondria, ER, Golgi apparatus, lysosomes, and nucleus. Therefore, the traditional model has recently been challenged, leading to new insights into the role of Drp1 in mitochondrial fission. Next, we highlight five of these main insights emerging in the literature suggesting directions for further research.

#### Drp1 localization to different organelles labels mitochondrial contraction sites to determine mitochondrial fate

A recent study revealed that different mitochondrial contraction sites lead to distinct mitochondrial fates [81]. Under physiological conditions, most healthy mitochondria undergo midzone fission (splitting in two at the middle), with contraction sites marked by replicating mtDNA. This process is mainly completed with the cooperation of Drp1, MFF, ER, and actin. Under pathological conditions such as ischemia and hypoxia, damaged mitochondria undergo peripheral fission (splitting from the ends, producing large and small segments), with the contraction sites marked by non-replicating mtDNA. This process involves Drp1, Fis1, and lysosomes [114] (Fig. 2①). In addition to Mito-Drp1, Drp1 distributed in the ER (ER-Drp1) and lysosomes (Lyso-Drp1) also participate in the labeling of mitochondrial contraction sites under physiological and hypoxic-ischemic conditions.

**Fig. 2:**
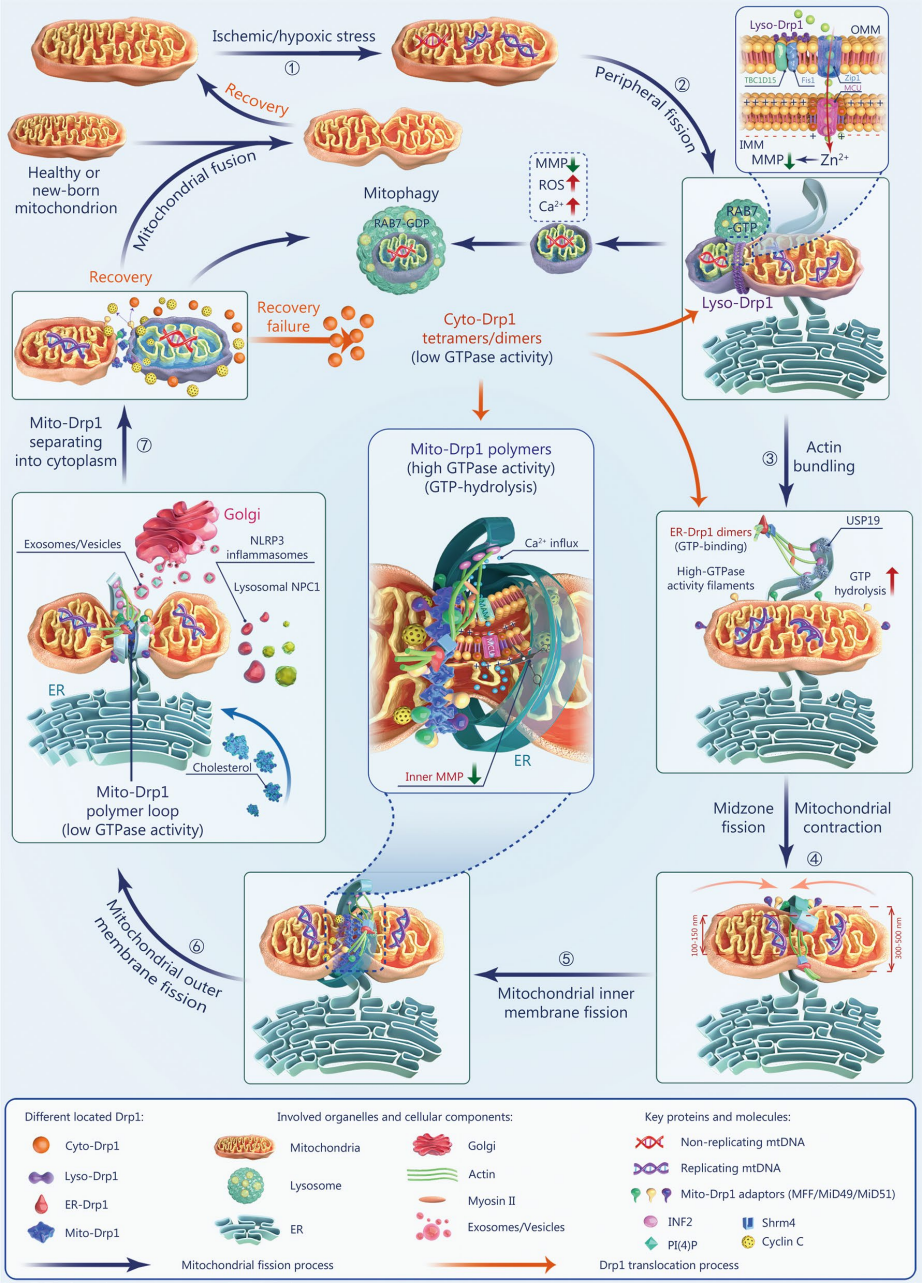
Schematic representation of Drp1-mediated multi-step processes during mitochondrial fission. **①** Emergence of mtDNA bad sectors. Damaged mitochondria undergo peripheral fission, whose contraction sites are marked by non-replicating mtDNA. **②** Lyso-Drp1 mediated mitochondrial peripheral fission. Lyso-Drp1 interacts with Zip1 to promote Zn^2+^ removal through the Zip1-MCU channel. **③** ER-Drp1 mediated-actin bundling. Activated ER-Drp1 dimers promote the efficiency of ER winding around mitochondria by aggregating actin into bundles through Shrm4 binding. **④** ER-Drp1-driven mitochondrial pre-contraction. Mito-Drp1 adapters migrate to the ER-Drp1-driven mitochondrial pre-contraction site. **⑤** Mito-Drp1-mediated IMM fission. Cyto-Drp1 aggregates into the Mito-Drp1 polymer loop at the ER-Mito contact site, destroying the MCU on the IMM before outer membrane fission. **⑥** Mito-Drp1 and Golgi-derived PI(4)P vesicle co-mediated MOM fission. Mito-Drp1 polymer loop further contracts and cleaves the MOM via GTP hydrolysis. The recruitment of Golgi-derived vesicles is involved in this process. **⑦** Mito-Drp1 separation into the cytoplasm. Mito-Drp1 separates from cyclin C and is reconverted to Cyto-Drp1 tetramers/dimers with low GTPase activity. Drp1 Dynamin-related protein 1, IMM inner mitochondrial membrane, MCU mitochondrial calcium uniporter, MOM mitochondrial outer membrane, TBC1D15 TBC domain family member 15, OMM outer mitochondrial membrane, Cyto-Drp1 cytoplasmic Drp1, Lyso-Drp1 lysosome Drp1, ER-Drp1 endoplasmic reticulum-Drp1, Mito-Drp1 mitochondrial Drp1, Fis1 mitochondrial fission protein 1, MMP mitochondrial membrane potential, ROS reactive oxygen species, NLRP3 NOD-, LRR- and pyrin domain-containing protein 3, ER endoplasmic reticulum, mtDNA mitochondrial DNA, MFF mitochondrial fission factor, INF2 inverted formin 2, Shrm4 shroom4, PI(4)P phosphatidylinositol 4-phosphate

Lyso-Drp1 can interact with the mitochondrial zinc transporter protein Zip1 to induce a transient decrease in membrane potential at the mitochondrial contraction site via Zn^2+^ removal through the Zip1-mitochondrial calcium uniporter (MCU) channel, facilitating recognition of the mitochondrial contraction site by subsequent Mito-Drp1 polymers [115, 116]. Additionally, a study on myocardial ischemia–reperfusion mouse model showed that TBC domain family member 15 (TBC1D15 and Rab7) are involved in Drp1-mediated mitochondrial-lysosomal contacts and subsequent mitochondrial peripheral fission [117] (Fig. 2②). These findings suggest that labeling of the mitochondrial contraction sites is critical for determining the manner of fission and the ultimate mitochondrial fate, which depends on the Drp1-mediated recruitment of different organelles through various protein interactions in the context of ischemia and hypoxia.

#### Drp1 functions prior to ER-Mito contact-induced mitochondrial contraction

Disruption of the ER-Mito contact does not affect Drp1 recruitment to the mitochondria [118], suggesting that Drp1 may not simply be passively recruited after ER-Mito contact-induced mitochondrial contraction.

ER-Drp1 dimers accelerate ER-Mito contact formation by inducing ER tubulation, independent of GTP hydrolysis by Drp1 [112]. We recently showed that Drp1 participates in ER-Mito contact formation prior to its recruitment to the contraction site under ischemia and hypoxia. Activated ER-Drp1 dimers promote the efficiency of ER winding around mitochondria by aggregating actin into bundles through shroom4 (Shrm4) binding (Fig. 2③). Zhao et al. [119] further visualized the involvement of Drp1 in ER-Mito contact formation in live cells.

During ER-Mito contact formation in ischemia and hypoxia, actin bundling induced by ER-Drp1 dimers further promotes Mito-Drp1 polymer loop formation and increases GTP hydrolysis by Mito-Drp1 [120, 121]. This mechanism is related to the de-ubiquitination of MOM proteins [122] and enhancement of actin-Mito-Drp1 polymer affinity by myosin II [123]. USP19, a de-ubiquitinating enzyme on the ER membrane, can initiate mitochondrial protein de-ubiquitination by binding FUNDC1 after aggregating to ER-Mito contacts, further enhancing GTP hydrolysis by Mito-Drp1 in ischemia and hypoxia [122] (Fig. 2③).

#### Drp1 participates in IMM fission by regulating mitochondrial matrix calcium

Before Mito-Drp1 polymer loops mediate the fission of the MOM, they participate in the contraction and fission of the IMM by modulating the calcium content in the matrix [124] (Fig. 2④). An increase in matrix calcium content leads to IMM contraction, which is possibly associated with altered electron transport chain activity [125, 126]. We previously showed that ischemia-induced Drp1 activation can block the formation of mitochondrial respiratory chain complex I, disrupting the electron transport chain [14]. This process may be related to the disruptive effect of Mito-Drp1 on MCU channels in the IMM [127] (Fig. 2⑤).

#### Golgi-derived vesicles are involved in Drp1-mediated MOM cleavage

The massive recruitment of Golgi-derived vesicles at mitochondrial contraction sites is believed to play a role in the Drp1-mediated cleavage of the MOM, and this process may critically involve vesicle-enwrapped phosphatidylinositol 4-phosphate [PI(4)P] [128]. Decreased production of PI(4)P leads to filamentous lengthening or no breakage in the MOM at contraction sites [128, 129]. NOD-, LRR- and pyrin domain-containing protein 3 (NLRP3) inflammasomes may also affect Drp1-mediated MOM cleavage by regulating phospholipid bilayer integrity and fluidity. PI(4)P on the Golgi can induce the disassembly of the trans-Golgi network by recruiting NLRP3 inflammasomes [130]. The lysosomal cholesterol transporter Niemann-Pick C1 protein (NPC1) inhibits inflammasome activation by blocking cholesterol transport to the ER [131]. These studies emphasize the significance of multi-organelle interactions and NLRP3 inflammasomes in the Drp1-mediated MOM cleavage in ischemia and hypoxia (Fig. 2⑥).

#### Nuclear cyclin C is involved in Mito-Drp1 aggregation and segregation

In ischemia and hypoxia, a large amount of nuclear cyclin C is released into the cytoplasm, which then bind Cyto-Drp1 tetramers/dimers with low GTPase activity and wind around contraction sites to form Mito-Drp1 polymer loops with high GTPase activity. When Mito-Drp1 polymers complete cleavage of the MOM via GTPase hydrolysis, Mito-Drp1 separates from cyclin C and is reconverted to Cyto-Drp1 tetramers/dimers with low GTPase activity [132] (Fig. 2⑦).

### Functions of Drp1 in mitochondrial bioenergetics

Drp1 typically regulates mitochondrial bioenergetics, including MMP, ATP production, ROS production, the tricarboxylic acid (TCA) cycle, mitochondrial respiration (oxidative phosphorylation), and metabolism. Under normal conditions, moderate Drp1 phosphorylation drives basal fission and ATP production, thus maintaining homeostasis. However, hypoxic-ischemic injury-induced changes of Drp1 oligomerization and modification dysregulate mitochondrial bioenergetics, including reduced MMP (ΔΨm), decreased ATP content, and excessive ROS [14, 16, 133].

#### Drp1-induced mitochondrial calcium overload in ischemia and hypoxia leads to decreased MMP

In ischemia and hypoxia, activated Drp1 disrupts the mitochondrial MCU by affecting mitochondrial calcium uptake 1 (MICU1) [127], which, along with calcium uptake receptors, such as Rapid Mode of mitochondrial calcium uptake (RaM) and mitochondrial ryanodine receptor (mRyR) [17, 134], causes massive calcium ion transport into the mitochondrial matrix, resulting in mitochondrial calcium overload (Fig. 3①). This can be compensated by activation of calcium release channels and Na^+^-H^+^ exchangers on the mitochondrial membrane. These include the mPTP [13] and mitochondrial sodium-calcium exchanger (mNCX) [135], whose activity can result in the accumulation of a large amount of H^+^ in the mitochondrial matrix, thus causing a decrease in the electrochemical gradient across the IMM, which is a key factor in Drp1-induced MMP reduction in ischemia and hypoxia (Fig. 3②). In hypoxemia caused by COVID-19, SARS-CoV-2 activates Drp1-induced mitochondrial calcium overload, leading to excessive opening of the mPTP [48], whereas blocking mPTP can enhance mitochondrial calcium retention capacity and bioenergetics [136].

**Fig. 3:**
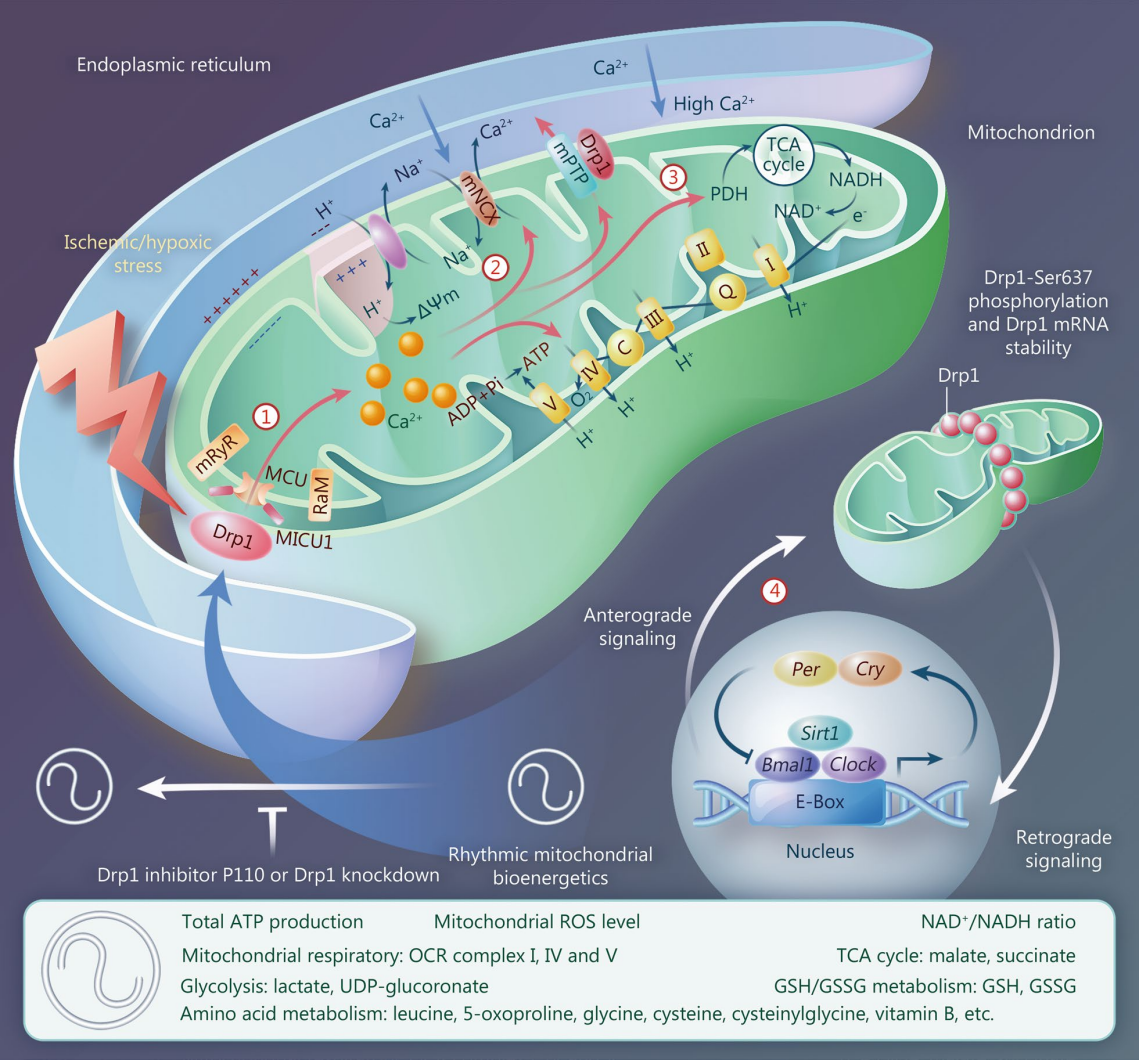
Schematic representation of Drp1 regulating multiple mitochondrial bioenergetic processes by inducing mitochondrial calcium overload in ischemia and hypoxia. ① Activated Drp1 induces mitochondrial calcium overload via MICU1/2, which disrupts the MCU channel. ② Drp1-induced mitochondrial calcium overload reduces mitochondrial membrane potential (ΔΨm) by promoting Na^+^-Ca^2+^ and Na^+^-H^+^ exchange at the IMM. ③ Drp1-induced mitochondrial calcium overload disrupts the TCA cycle and oxidative phosphorylation by affecting PDH activity, resulting in decreased ATP production and increased consumption. ④ “Circadian clock” effect of Drp1-Ser637 phosphorylation influences rhythmic mitochondrial bioenergetics, which may be related to the transcription-enzymatic interplay of rhythm genes *Bmal1*/*Clock*, and *Drp1*. Drp1 Dynamin-related protein 1, IMM inner mitochondrial membrane, TCA tricarboxylic acid, PDH pyruvate dehydrogenase, MCU mitochondrial calcium uniporter, MICU1 mitochondrial calcium uptake 1, RaM rapid Mode of mitochondrial calcium uptake, mRyR mitochondrial ryanodine receptor, ADP adenosine diphosphate, Pi inorganic phosphate, ATP adenosine triphosphate, NCX sodium-calcium exchanger, mPTP mitochondrial permeability transition pore, Per period, Cry cryptochrome, ROS reactive oxygen species, OCR oxygen consumption rate, UDP uridine diphosphate, GSH glutathione, GSSG glutathione disulfide

#### Drp1-induced mitochondrial calcium overload in ischemia and hypoxia leads to a decrease in cellular ATP content

In ischemia and hypoxia, considerably more ATP is consumed owing to mitochondrial calcium overload and proton exchange induced by Drp1, which does not balance the rate of ATP generation by the compensatory effect of mitochondrial fission and increased mitochondrial number. Moreover, the large amount of cytoplasmic calcium released from the ER causes approximately 1000-fold increase in mitochondrial calcium uptake [137], which affects pyruvate dehydrogenase (PDH) activity and substrate metabolism, while disrupting the TCA cycle and oxidative phosphorylation [14], resulting in an overall decrease in ATP production (Fig. 3③). Thus, Drp1 activation in ischemia and hypoxia leads to mitochondrial calcium overload accompanied by downregulated ATP levels and ultimately causes considerable ROS accumulation.

#### Rhythmic fluctuations of Drp1 activity are responsible for the “circadian clock” effect of mitochondrial bioenergetics

Mitochondrial bioenergetics exhibit circadian regulation that is possibly driven by rhythmic variation in Drp1 phosphorylation at Ser637 [49], as observed for ATP content, mitochondrial ROS (not total ROS), mitochondrial respiration [e.g., oxygen consumption rate (OCR)], and various mitochondrial metabolites (e.g., glutathione, glycine, lactate, malate, and succinate).

Rhythmic fluctuations of Drp1 activity regulate mitochondrial bioenergetics via rhythmic changes in complex I, IV, and V levels [14, 138] through various potential mechanisms, including maintaining the NAD^+^/NADH ratio, mitochondrial SIRT1 activity, mitochondrial protein acetylation [139], and mitochondrial morphology [49]. Although the transcriptional regulatory mechanism affecting rhythmic changes in Drp1 activity remains unknown, it may be related to the transcription-enzymatic interplay of the rhythm genes *Bmal1*/*Clock* and *Drp1* [140] (Fig. 3④).

### Drp1 functions in mitophagy

Mitophagy is a selective form of autophagy that maintains intracellular homeostasis by degrading dysfunctional mitochondria under various pathological conditions, such as ischemia and hypoxia, energy deficiency, and oxidative stress, which is vital for cell survival and functional maintenance [141, 142]. Drp1-mediated mitophagy is closely related to mitochondrial fission. Mitophagy-related proteins, such as PINK1, Parkin, FUN14 domain-containing protein 1 (FUNDC1), and BCL2-like 13 (BCL2L13), regulate Drp1-mediated mitochondrial fission [143]. FUNDC1 can bind Drp1 to initiate mitochondrial fission before being recruited to the ER-Mito contact site to interact with calnexin and initiate mitophagy [144]. Thus, FUNDC1 may synergistically regulate Drp1-mediated mitophagy and fission. Interfering with PINK1 upregulates Drp1-Ser616 phosphorylation, thereby accelerating mitochondrial fission and damage [145], while *Drp1* knockdown does not affect mitochondrial PINK1 expression [11]. Drp1 may therefore be a downstream target of PINK1 [146]. These observations suggest that Drp1-mediated mitophagy and mitochondrial fission are mutually reinforcing and relatively independent processes. Whether the two act synergistically or antagonistically likely depends on the degree of ischemic-hypoxic damage that can be tolerated.

#### Synergistic effects of Drp1-mediated mitophagy and mitochondrial fission in moderate ischemia and hypoxia

**Fig. 4:**
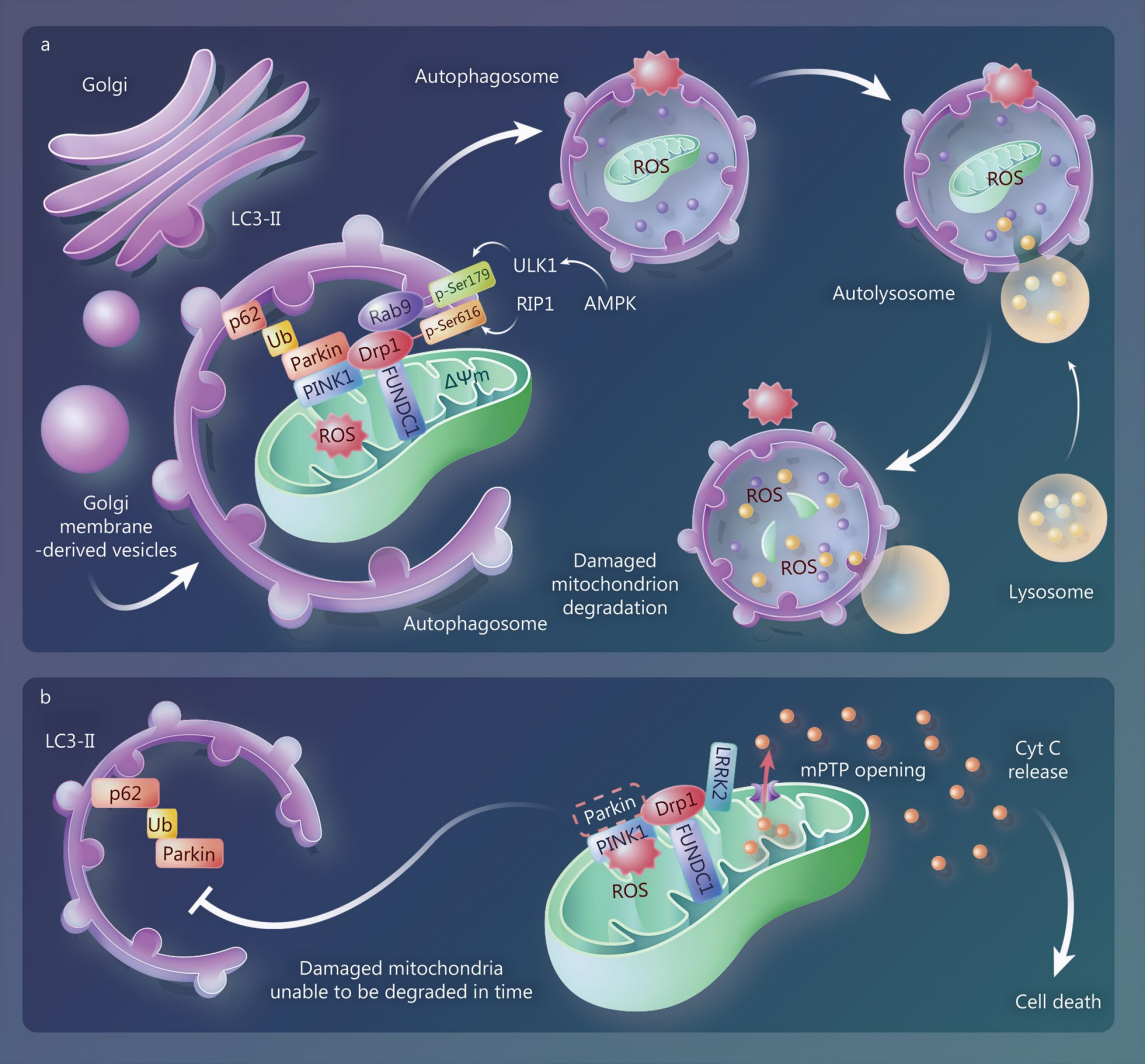
Effects of Drp1 on mitophagy are closely related to the degree of ischemia and hypoxia. **a** Moderate ischemia/hypoxia-induced compensatory mitochondrial damage. In moderate ischemia/hypoxia, AMPK activates ULK1 and phosphorylates Rab9 at Ser179, which promotes the association between Rab9 and RIP1, followed by Drp1 phosphorylation at Ser616. Damaged mitochondria are sequestered by autophagosomes derived from the trans-Golgi network. **b** Severe ischemia/hypoxia-induced decompensated mitochondrial damage. In severe ischemia/hypoxia, mitochondrial Drp1 recruits LRRK2, thereby preventing Parkin translocation and reducing the encapsulation and degradation of damaged mitochondria by autophagosomes. Drp1 Dynamin-related protein 1, AMPK adenosine monophosphate-activated protein kinase, ULK1 unc-51-like kinase 1, LRRK2 leucine-rich repeat kinase 2, LC3-II microtubule-associated protein 1B-light chain 3, Ub ubiquitin, PINK1 PTEN-induced kinase 1, ROS reactive oxygen species, FUNDC1 FUN14 domain-containing protein 1, RIP1 receptor interacting protein 1, mPTP mitochondrial permeability transition pore, Cyt C cytochrome C

In moderate hypoxic-ischemia-induced mitochondrial depolarization, Drp1 and Parkin are co-recruited to the MOM, adjacent to PINK1 [147]. The unc-51-like kinase 1 (ULK1)-Rab9-RIP1-Drp1 protein complex is involved in mitophagy, with ULK1-induced Rab9 activation on Golgi membranes and binding to RIP1-activated Drp1 on the damaged MOM [148]. Activated adenosine monophosphate-activated protein kinase (AMPK) promotes Drp1-mediated mitochondrial fission and mitophagy via ULK1 phosphorylation [149]. Disruption of the Rab9-Drp1 complex inhibits the formation of mitochondrial autophagic vesicles so that damaged mitochondria cannot be cleared and myocardial ischemic injury is exacerbated [148]. Meanwhile, moderate ischemic preconditioning can activate ULK1 and trigger FUNDC1-Drp1-mediated mitophagy, exerting a renoprotective effect [150] (Fig. 4a). These findings suggest that Drp1-mediated mitophagy and mitochondrial fission are synergistically upregulated in moderate hypoxic-ischemia-induced compensatory mitochondrial injury and are crucial for cellular homeostasis and preventing further tissue damage.

#### Antagonistic effects of Drp1-mediated mitophagy and mitochondrial fission in severe ischemia and hypoxia

In mitochondrial decompensated injury caused by severe ischemia and hypoxia, Mito-Drp1 binds LRRK2, causing excessive opening of mPTP channels on the MOM and accelerating cell death. LRRK2 can also disrupt the interaction between Mito-Drp1 and Parkin, inhibiting mitophagy [151]. In *Drp1* knockout or Drp1 K38A HeLa cells, loss of Drp1 promotes Parkin recruitment by mitochondrial PINK1 and increases mitophagy [152], confirming that Drp1 activation inhibits Parkin-mediated mitophagy in severe ischemia and hypoxia [11]. Thus, binding of Mito-Drp1 to LRRK2 may be stronger than that of Drp1 to Parkin in severe ischemia and hypoxia, leading to active or passive segregation of Parkin in the cytoplasm, which ultimately inhibits Drp1-mediated mitophagy (Fig. 4b).

In summary, if mitochondria can withstand moderate ischemic-hypoxic injury and undergo self-repair, mitophagy is activated to clear damaged mitochondria, maintaining homeostasis and thus preventing cell death and tissue damage. However, if mitochondria cannot withstand severe ischemic-hypoxic injury and decompensation occurs, mitophagy is inhibited and the large number of damaged mitochondria produced by excessive mitochondrial fission cannot be removed effectively. Further research is needed to define the tolerance to ischemic-hypoxic injury and identify regulatory receptors that could determine whether mitophagy is enhanced or inhibited under different degrees of ischemic-hypoxic injury.

### Functions of Drp1 in cell death

Cell death processes, including apoptosis, necroptosis, pyroptosis, and ferroptosis, have been linked to changes in Drp1 activity and expression [153].

#### Drp1-Bcl-2 associated X (BAX) apoptosis positive feedback regulation loop

In ischemia and hypoxia, Drp1 can interact with BAX to cause apoptosis [11, 154]. BAX induces Drp1 SUMOylation and alters its physicochemical properties [155]. In turn, SUMOylated Drp1 further activates the BAX-mediated apoptotic pathway, resulting in a positive feedback regulation mechanism of Drp1-BAX activation [56].

Mito-Drp1 accelerates BAX translocation from the cytoplasm to ER-Mito contact sites in hypoxic-ischemic injury and physically binds to mitochondrial BAX. This causes excessive opening of the mPTPs, leading to increased cytochrome C release and caspase-9/-3 activation, thus accelerating the apoptotic process [11]. The mitochondrial translocation of BAX occurs later than that of Drp1 [156, 157], further confirming that BAX is recruited by Mito-Drp1 before initiation of the above-mentioned events. In addition, the excessive opening of mPTP further disrupts permeability of the outer mitochondrial membrane, inducing the release of the IMM protein DDP/TIMM8a (deafness dystonia peptide/translocase of inner mitochondrial membrane 8a), which binds to Cyto-Drp1 and promotes mitochondrial fission. This forms a positive feedback loop of the mitochondrial fission-apoptosis mechanism [158] (Fig. 5①).

**Fig. 5:**
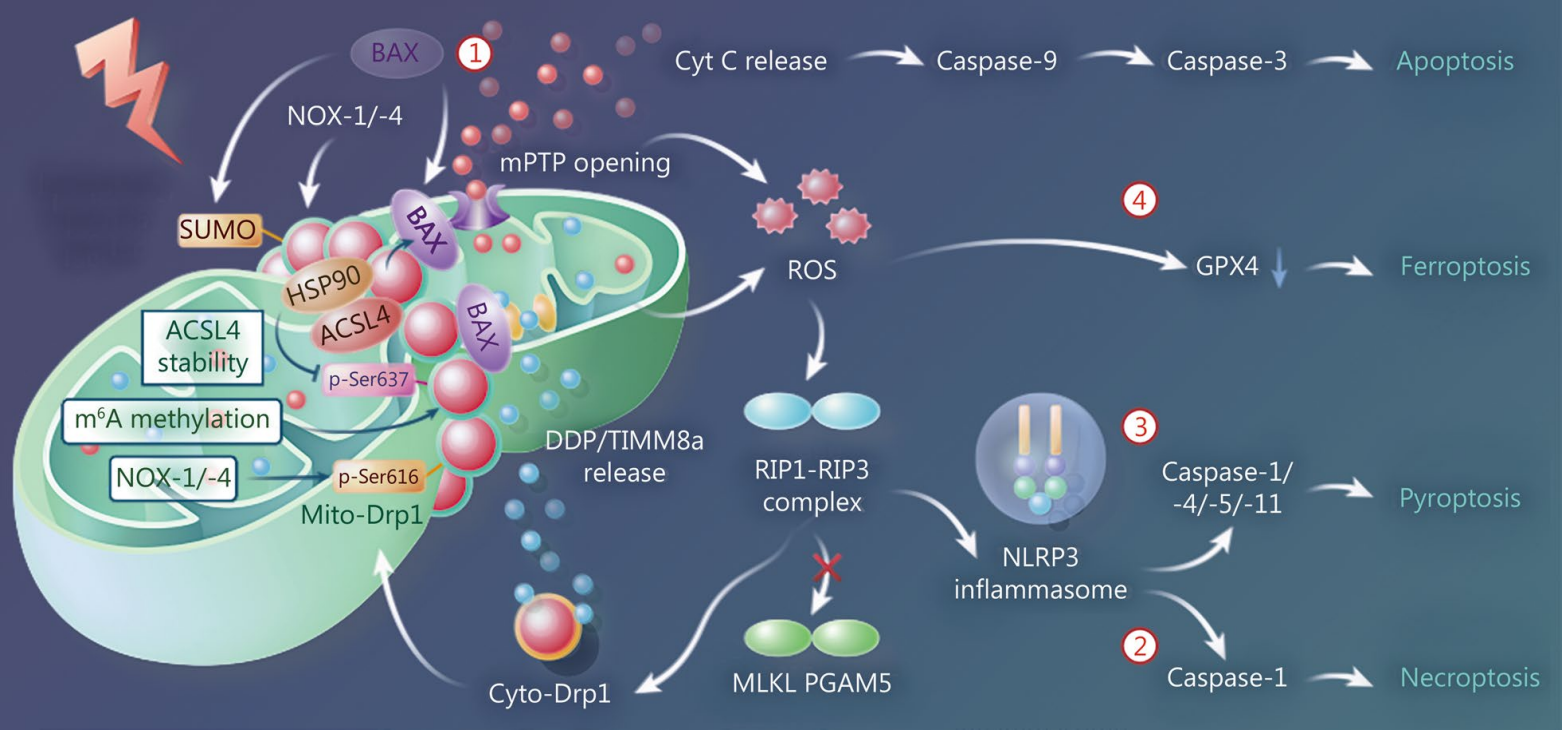
Drp1 is involved in the regulation of multiple cell death processes in ischemia and hypoxia. ① Drp1-BAX apoptosis-positive feedback regulation loop. ② RIP1-RIP3-Drp1 necroptosis pathway. ③ Drp1-NLRP3 pyroptosis pathway. ④ HSP90-Drp1-ACSL4-GPX4 ferroptosis pathway. Drp1 Dynamin-related protein 1, SUMO SUMOylation, HSP90 heat shock protein 90, NOX-1/-4 NADPH oxidase 1 and 4, mPTP mitochondrial permeability transition pore, DDP/TIMM8a deafness dystonia peptide/translocase of inner mitochondrial membrane 8a, Cyto-Drp1 cytoplasmic Drp1, Cyt C cytochrome C, ROS reactive oxygen species, RIP1 receptor interacting protein 1, MLKL mixed lineage kinase domain-like protein, PGAM5 phosphoglycerate mutase 5, NLRP3 NOD-, LRR- and pyrin domain-containing protein 3, GPX4 glutathione peroxidase 4

In acute myocardial ischemia, Sirtuin 3 (SIRT3) downregulation promotes Drp1 activation, thereby activating caspase-9 and triggering apoptosis in cardiomyocytes to accelerate myocardial fibrosis and inflammation [159]. In hepatic ischemia–reperfusion, the ubiquitin protease ubiquitin-specific protease 15 (USP15) can stabilize oxidoreductase p66Shc expression, accelerating both Drp1-mediated mitochondrial fission and apoptotic processes [160]. Inhibition of Drp1 activity [161] or accelerated degradation of ubiquitinated Drp1 [162] can significantly suppress apoptosis.

#### RIP1-RIP3-Drp1-mediated necroptosis

Necroptosis is regulated by various kinases, such as RIP1, RIP3, mixed lineage kinase domain-like protein (MLKL), and PGAM5 [163]. Excessive mPTP opening is a prerequisite for necroptosis [13, 164], and Drp1-mediated ROS accumulation is a major trigger of necroptosis in ischemia and hypoxia [16, 165]. In myocardial ischemia–reperfusion, Drp1 translocation to the mitochondria promotes necroptosis in a time-dependent manner [166]. The RIP1-RIP3-Drp1 pathway is a classic NLRP3 inflammasome activation pathway during necroptosis, while MLKL and PGAM5 are not involved in this process [167] (Fig. 5②).

#### Drp1 in pyroptosis and ferroptosis

Although studies on Drp1 in pyroptosis and ferroptosis during ischemia and hypoxia are limited, their importance cannot be overlooked. Pyroptosis is a newly discovered mode of programmed cell death characterized by dependence on inflammatory caspases (mainly caspase-1, -4, -5, and -11) accompanied by the release of pro-inflammatory factors. Li et al. [168] showed that Drp1 plays a vital role in myocardial pyroptosis, with NADPH oxidase 1 (NOX-1) and NOX-4 inducing Drp1 activation and leading to NLRP3-mediated myocardial cell pyroptosis. In hypoxic pulmonary hypertension, m^6^A-induced FOXF1 adjacent non-coding developmental regulatory RNA (FENDRR) degradation promotes pyroptosis by regulating Drp1 promoter methylation in pulmonary artery endothelial cells [169] (Fig. 5③). Ferroptosis is an iron-dependent, caspase-independent form of cell death. Miao et al. [170] showed that heat shock protein 90 (HSP90) can induce Drp1-Ser637 dephosphorylation and form a HSP90-Drp1 complex, which, in turn, binds acyl-coenzyme A synthetase long-chain family member 4 (ACSL4) and stabilizes its expression. Upregulation of the HSP90-Drp1-ACSL4-complex reduces glutathione peroxidase 4 (GPX4) activity and accelerates ferroptosis by increasing lipid ROS production and mitochondrial fragmentation [170] (Fig. 5④).

### Role of Drp1 in cytoskeleton stability

**Fig. 6:**
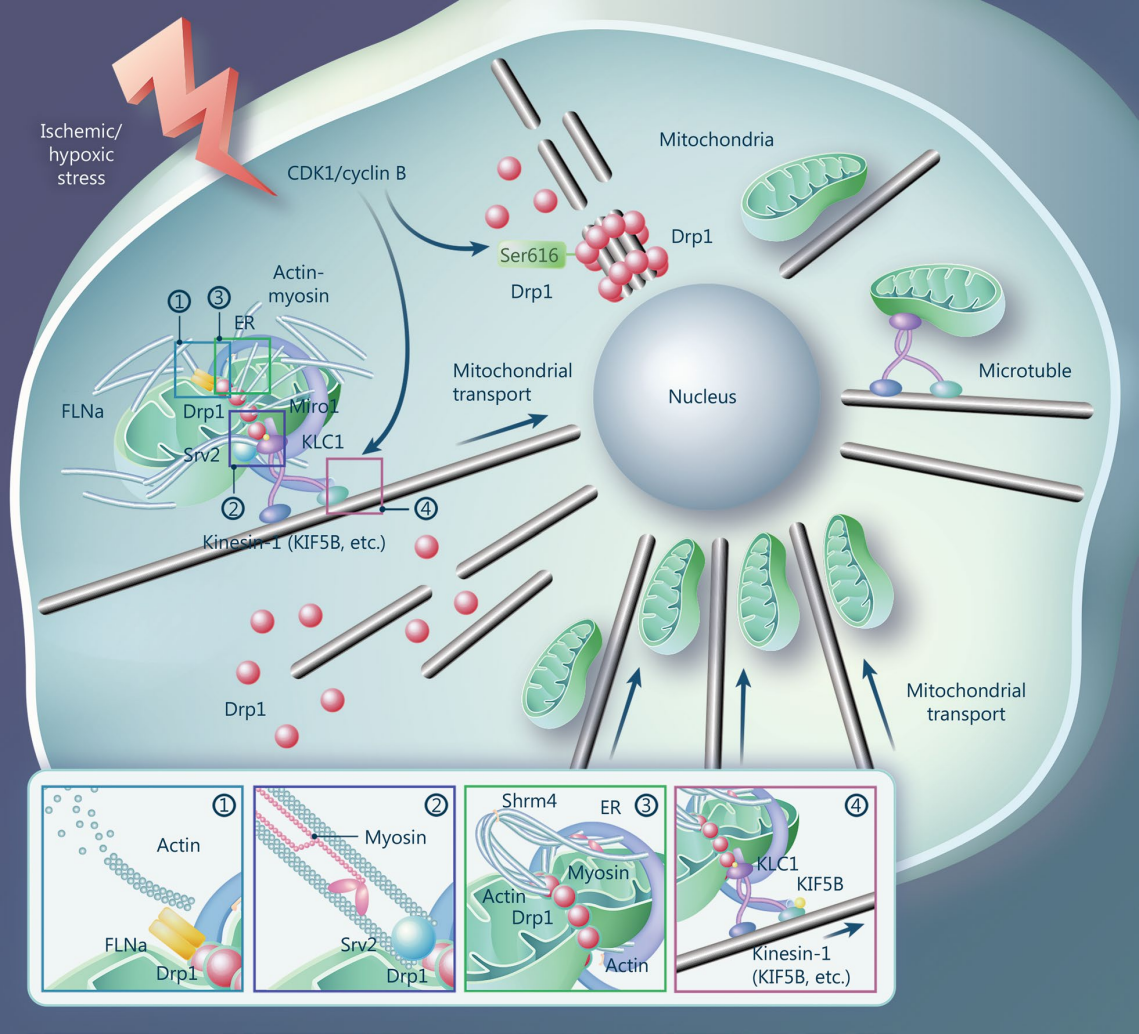
Drp1 participates in mitochondrial fission and transport by regulating cytoskeleton stability in ischemia and hypoxia. The GTPase domain of Drp1 interacts with FLNa to promote actin aggregation. Shrm4-bound activated Drp1 induces actin bundling to form ER-Mito contacts. The Srv2/Drp1 interaction facilitates Srv2-mediated modulation of actin polymerization on mitochondria. CDK1/cyclin B contributes to Drp1 dissociation from microtubules. Drp1-KLC1 coupling triggers KIF5B displacement from the kinesin-1 complex, increasing its binding to microtubule tracks and, thus, mitochondrial transport. High Drp1 levels exacerbate this mechanism, leading to the repositioning of mitochondria closer to the nucleus after ischemic/hypoxic stress. Drp1 Dynamin-related protein 1, FLNa filamin A, Shrm4 shroom4, ER-Mito endoplasmic reticulum and mitochondria, Srv2 suppressor of Rho3, CDK1 cyclin-dependent kinase 1, KLC1 kinesin light chain 1, KIF5B kinesin family member 5B, ER endoplasmic reticulum, Miro1 mitochondrial Rho GTPase 1

Drp1-mediated regulation of the cytoskeleton (e.g., actin, microtubule) is essential for mitochondrial dynamic changes and pathogenesis. In ischemic cardiomyopathy, the Drp1 GTPase domain interacts with the actin-binding cytoskeleton protein filamin A to promote actin aggregation and increase mitochondrial fission in cardiomyocytes [171]. Experiments with hypoxia-treated vascular smooth muscle cells suggested that Shrm4-bound activated Drp1 may induce actin bundling and then wrap the ER around mitochondria via inverted formin 2 (INF2) to form ER-Mito contacts. In yeast, deletion of actin regulatory protein suppressor of rho1 vacuole 2 (Srv2) results in abnormal actin assembly, leading to excessive mitochondrial lengthening or fusion and reduced mitochondrial respiratory capacity. Drp1 recruitment to mitochondria, which regulates actin assembly and its Srv2 interactions, alters mitochondrial fission and mitochondrial respiration [172] (Fig. 6). These studies suggest that Drp1-regulated actin aggregation plays a crucial role in mitochondrial fission.

Drp1 also exerts a regulatory effect on microtubules. Under normal conditions, Drp1 localizes on microtubule bundles to form kinetic aggregates that maintain microtubule stability and prevent mitochondrial fragmentation. Phosphorylation of Drp1 at Ser616 by CDK1/cyclin B separates Drp1 from microtubules, promoting mitochondrial fission through specific splicing [173]. In addition, Drp1 is involved in mitochondrial transport along the microtubules. Drp1 can also be conjugated to kinesin light chain 1 (KLC1) to trigger kinesin family member 5B (KIF5B) displacement from the kinesin-1 complex, which is involved in the microtubule-dependent transport of mitochondria [174] (Fig. 6). In acute ischemic-hypoxic injury, Drp1 activation accelerates this process, leading to mitochondrial aggregation along the microtubules toward the nucleus and destabilizing the mitochondrial network. Interference with Drp1 can restore normal mitochondrial distribution by reducing kinesin-1 activity [174].

### Functions of Drp1 in vesicle release and formation

**Fig. 7:**
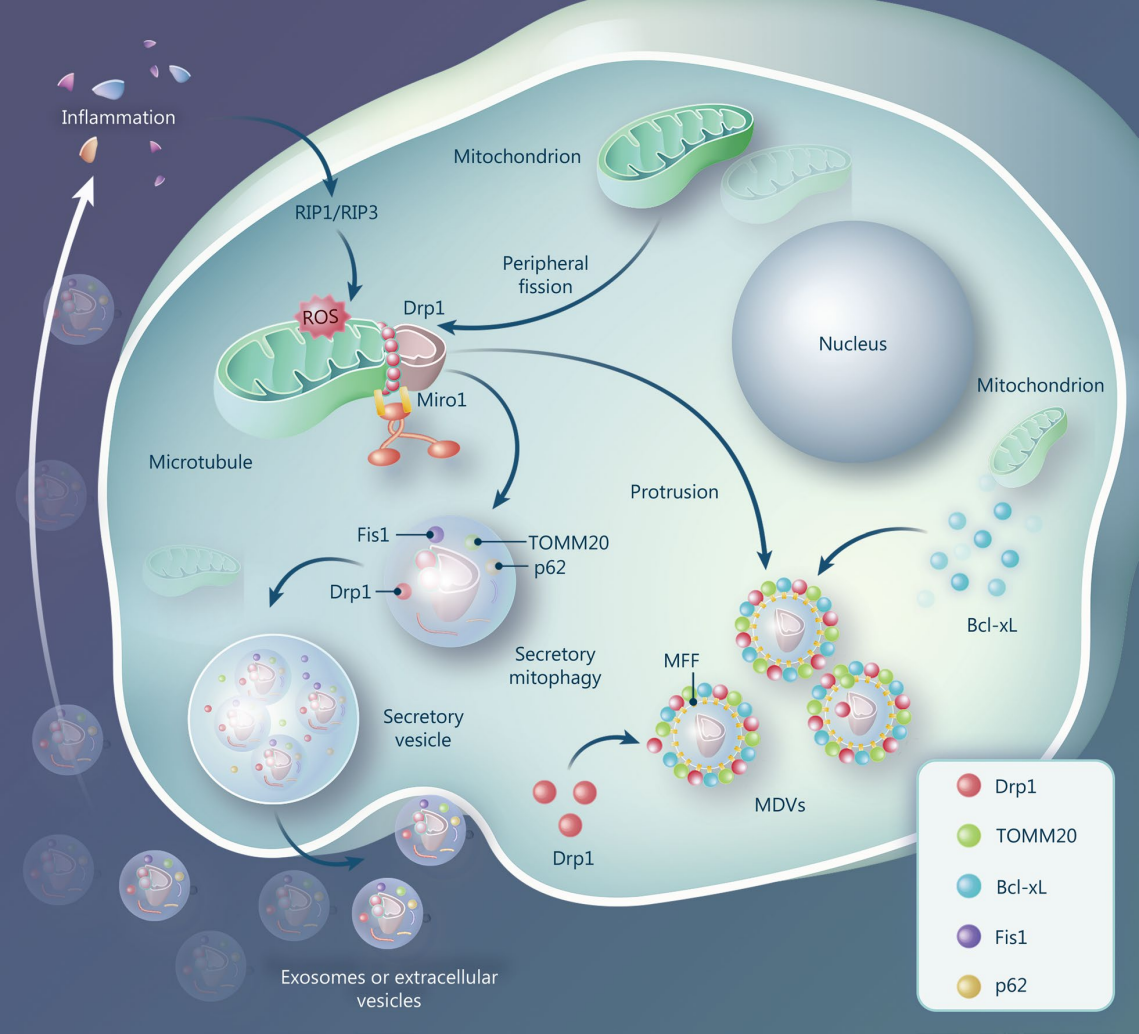
Drp1 is involved in the formation and release of vesicles in ischemia and hypoxia. Drp1-mediated mitochondrial quality imbalance stimulates vesicle secretion and inflammatory cascade activation. MFF on vesicle membranes can recruit cytoplasmic Drp1 to interact with Bcl-xL, resulting in changes in vesicle membrane fluidity. Drp1-binding vesicles can shed Miro1-induced mitochondrial membrane embossments and transform into MDVs. Drp1 dynamin-related protein 1, RIP1 receptor interacting protein 1, ROS reactive oxygen species, Miro1 mitochondrial Rho GTPase 1, Fis1 mitochondrial fission protein 1, TOMM20 translocase of outer mitochondrial membrane 20, MFF mitochondrial fission factor, Bcl-xL B-cell lymphoma-extra large, MDVs mitochondrial-derived vesicles

Mitochondria-associated vesicle formation, mitochondrial dynamics, and mitophagy synergistically maintain mitochondrial quality and cellular homeostasis [175, 176]. Mitochondrial-related proteins, such as Drp1, Fis1, sequestosome 1 (p62), PINK1, and translocase of outer mitochondrial membrane 20 (TOMM20), have been detected in secreted vesicles [16, 177]. Moderate ischemia and hypoxia-induced compensatory mitochondrial damage increases the formation of vesicles encapsulating damaged mitochondria, which can selectively clear damaged parts without affecting other organelles [176]. Severe ischemia and hypoxia-induced decompensatory mitochondrial damage activates Mito-Drp1 to accelerate p62-mediated mitophagosome formation and prevent the transformation of mitophagosomes to mitolysosomes via the RIP1-RIP3 pathway. Non-degraded mitophagosomes are secreted extracellularly in the form of vesicles to trigger inflammatory cascades, forming a detrimental cycle [16]. We observed a similar phenomenon in an acute myocardial ischemia model [133], suggesting that Drp1-mediated mitochondrial imbalances stimulate vesicle secretion and inflammatory cascade activation, especially in severe ischemic-hypoxic injury (Fig. 7).

Drp1 not only affects vesicle release via mitophagy but also directly impacts vesicle formation after ischemia and hypoxia [178]. MFF, distributed on vesicle membranes, can recruit Cyto-Drp1 to interact with B-cell lymphoma-extra large (Bcl-xL), resulting in changes in vesicle membrane fluidity [178]. Drp1-binding vesicles can shed mitochondrial membrane embossments induced by mitochondrial Rho GTPase 1 (Miro1) and transform into mitochondrial-derived vesicles (MDVs) [179] (Fig. 7). This is essential for mitochondrial quality control. However, the specific mechanisms require further in-depth exploration.

## Experimental compounds and clinical drugs targeting Drp1

Having explored the intricate molecular interactions and regulatory mechanisms of Drp1 in ischemia and hypoxia, it becomes clear that Drp1 plays a pivotal role in hypoxia/ischemia-induced mitochondrial quality imbalance. Targeting the oligomeric changes, modification activity, and protein interactions of Drp1 may open up potential avenues for therapeutic intervention to various hypoxic-ischemic diseases.

### Mitochondrial division inhibitor-1 (Mdivi-1)

Mdivi-1 is a small-molecule compound that can cross the blood–brain barrier. Mdivi-1 reduces Cyto-Drp1 translocation to the MOM by inhibiting Drp1 activity without affecting the expression of Drp1. Mdivi-1 allosterically binds Drp1, preventing its self-assembly and GTP hydrolysis [180]. In hypoxic and lipopolysaccharide-stimulated microglia, pretreatment with Mdivi-1 (25 μmol/L) for 12 h significantly downregulated Drp1-Ser616 phosphorylation and ROS levels [181]. In a rat model of cardiac arrest, Mdivi-1 administration (1.2 mg/kg) after the restoration of spontaneous circulation significantly protected against cerebral ischemic injury by inhibiting Drp1 activity-dependent mitochondrial fission and apoptosis pathways [182]. Therefore, pharmacological targeting of mitochondrial fission by Mdivi-1 may be a promising therapy for cardiac arrest [183]. In angiotensin-II-induced hypertension, Mdivi-1 treatment reversed angiotensin-II-induced Drp1 phosphorylation, restored mitochondrial activity, and reduced the phenotypic conversion of vascular smooth muscle cells, leading to low blood pressure [184]. In the treatment of subarachnoid hemorrhage, Mdivi-1 reduces neuroinflammation and neuronal apoptosis [185]. In diabetic combined myocardial ischemia–reperfusion injury, Mdivi-1 promotes mitochondrial fusion and attenuates cardiac mitochondrial dysfunction and aberrant dynamics, thereby reducing the infarct size [186].

Recent studies have revealed that Mdivi-1 also has Drp1-independent properties. In a clinically-relevant large animal model of acute myocardial ischemia, Mdivi-1 inhibits mitochondrial respiration and reduces ROS production in the absence of Drp1, without altering mitochondrial morphology [187]. Besides, Mdivi-1 does not appear to affect the mitochondrial translocation of BAX [156], while *Drp1* knockdown significantly inhibited this process in ischemia and hypoxia [11]. Additionally, the effect of Mdivi-1 on mitochondrial OCR could not be simulated by Drp1 knockdown [188]. These studies indicate that the effects of Mdivi-1 are not fully consistent with Drp1 inhibition, suggesting that Mdivi-1 may not be a specific inhibitor of Drp1.

Our recent study [15] revealed that the protective effect of Mdivi-1 on multi-organ functions in ischemic-hypoxic injury may be related to a reduction in nuclear factor erythroid 2-related factor 2 (Nrf2)-induced antioxidant enzymes. Moreover, a study of acute myocardial ischemia [189] revealed that the protective effects of Mdivi-1 on cardiac function are mediated via heme oxygenase 1 (Hmox1) upregulation to inhibit oxidative stress, in addition to inhibiting Drp1-mediated mitochondrial fission to attenuate cardiac fibrosis. These studies confirmed that the Drp1-independent properties of Mdivi-1 are closely related to its ability to target multiple oxidative stress pathways.

However, Mdivi-1 has toxic side effects that may hinder its clinical application. Mdivi-1 may induce mitochondrial depolarization and calcium depletion in the ER, thereby sensitizing oligodendrocytes to excitotoxicity and ER stress [190]. Furthermore, Mdivi-1 may cause severe developmental arrest of preimplantation embryos in a dose-dependent manner [191]. Thus, the therapeutic efficacy and toxicity of Mdivi-1 as a therapeutic for organ protection following hypoxic-ischemic injury should be comprehensively evaluated in future clinical studies.

### Peptide inhibitor (P110)

P110, a peptide inhibitor of Drp1 protein interactions, can improve mitochondrial function and reduce oxidative stress by disrupting the Drp1-LRRK2 interaction [101, 192]. P110 treatment significantly inhibited the mitochondrial translocation of Drp1 and reduced mitochondrial fragmentation in hypoxia-induced SH-5YSY cells [192], with similar effects observed in vivo [193].

Recent studies have also revealed that P110 treatment can inhibit the Drp1-Fis1 interaction, augmenting the activity of mitochondrial cytochrome C oxidase subunit I (COX I) and COX IV [101, 194, 195]. In cerebral ischemia–reperfusion, P110 encapsulated by macrophage-derived vesicles can reduce astrocyte mitochondrial damage by inhibiting the Drp1-Fis1 interaction, enabling the release of mitochondria from healthy astrocytes and their subsequent delivery to neurons, thus alleviating neuronal damage in ischemia and hypoxia [195]. Use of P110 to block the Drp1-Fis1 interaction in septic cardiomyopathy attenuated oxidative stress and reduced MMP in hypoxia and lipopolysaccharide-induced cardiomyocytes [194]. In colitis, systemic delivery of P110 can reduce intestinal inflammation by maintaining mitochondrial homeostasis in enterocytes and macrophages [196].

No side effects of P110 have been reported. As a peptide-like inhibitor that reduces mitochondrial fission by blocking Drp1 interactions, P110 has greater translation prospects than Mdivi-1, which reduces fission by inhibiting Drp1 activity.

### Mitoquinone (MitoQ)

MitoQ, an antioxidant that targets mitochondria, reduces mitochondrial fragmentation by inhibiting Drp1 translocation to the mitochondria [197]. MitoQ treatment also inhibits the mitochondrial translocation of BAX in hypoxic cells, in addition to upregulating other mitochondrial dynamics-related proteins, such as MFN1, MFN2, OPA1, and peroxisome proliferator-activated receptor gamma coactivator 1 (PGC1) [198], and improving MMP and ROS regulation. Furthermore, MitoQ can inhibit COX I, III, and IV activity, thereby decreasing proton pumping and oxygen consumption [199].

MitoQ exhibits protective effects against acute hypoxia-induced pulmonary dysfunction [200]. MitoQ can prevent Drp1-mediated mitochondrial fission, suppressing acute-hypoxia induced apoptosis and ROS generation in alveolar epithelial cells [161]. However, MitoQ does not affect the progression of chronic hypoxia-induced pulmonary hypertension [200], which may be related to varied Drp1 activity and ROS content at different stages of hypoxia.

MitoQ can improve vascular endothelial function and blood perfusion in patients with hypoxic-ischemic diseases [201, 202]. Long-term supplementation with MitoQ may reduce oxidative stress and improve cardiovascular function in older patients [202]. Moreover, MitoQ may reduce the risk of cardiovascular disease by augmenting nitric oxide signaling in hypoxia during pregnancy [201]. MitoQ can also attenuate secondary brain injury, accelerate hematoma regression by inhibiting NLRP3 inflammasomes, and promote microglial polarization in cerebral hemorrhage [203]. Additionally, MitoQ has been used as an organ-protective fluid for donor ischemic kidneys in renal transplantation, with significant effects in improving renal ischemia–reperfusion injury [204]. Currently, MitoQ has passed phase 2 clinical trials (Table 1) and has great potential for clinical application.

**Table 1:** The clinical trials for drugs targeting Drp1

NCT number	Phases	Enrollment	Study status	Interventions/Treatments	Conditions/Diseases
NCT02966665	Phase 1	420	Recruiting	MitoQ, BQ-123, BH4/acetylcholine, sodium nitroprusside, angiotensin-II, norepinephrine, phentolamine/angiotensin-II, valsartan/fexofenadine, ranitidine/BQ-123/BH4, L-NMMA, vitamin C, vitamin E, α-lipoic acid and L-ascorbate/maximum exercise	Chronic obstructive pulmonary disease, pulmonary artery hypertension, heart failure, hypertension
NCT04276740	Phase 2	206	Not yet recruiting	MitoQ/placebo	Ulcerative colitis flare
NCT00329056	Phase 2	128	Completed	MitoQ	Parkinson's disease
NCT03586414	NA	60	Recruiting	MitoQ, then placebo/placebo, then MitoQ	Diastolic dysfunction
NCT04267926	Phase 1/Phase2	60	Recruiting	20 mg MitoQ/40 mg MitoQ/placebo	Multiple sclerosis, fatigue
NCT03166800	Phase 1/Phase 2	9	Terminated	MitoQ/placebo	Multiple Sclerosis, Fatigue
NCT04098510	NA	10	Unknown	MitoQ	Healthy
NCT04109820	NA	15	Recruiting	MitoQ	Sickle cell disease
NCT03506633	NA	13	Recruiting	MitoQ	Peripheral arterial disease, peripheral artery disease
NCT04026711	Phase 1	40	Recruiting	MitoQ/placebo oral tablet	Asthma, obesity
NCT05539625	Phase 2	120	Not yet recruiting	MitoQ/placebo	Ulcerative colitis
NCT05561556	NA	60	Recruiting	MitoQ	Cardiovascular diseases, hypertension, racism, vascular diseases
NCT05686967	Phase 1	50	Recruiting	MitoQ/placebo/aerobic exercise	Aging, menopause
NCT02597023	NA	55	Completed	MitoQ/placebo	Aging
NCT05872139	NA	23	Completed	MitoQ/placebo	Aging, endothelial dysfunction, cardiovascular function, arterial stiffness
NCT04851288	Phase 2	112	Recruiting	MitoQ/placebo	Aging
NCT04334135	NA	60	Recruiting	MitoQ	Racial disparities, blood pressure, cardiovascular risk factor, renal function
NCT02364648	Phase 4	24	Unknown	MitoQ/placebo	Chronic kidney disease
NCT05605548	NA	24	Recruiting	MitoQ/placebo	Chronic obstructive pulmonary disease
NCT03960073	NA	25	Completed	MitoQ/placebo	Renal insufficiency, chronic, heart failure with preserved ejection fraction
NCT05146843	NA	44	Not yet recruiting	MitoQ/placebo	Breast neoplasms
NCT04558190	NA	10	Completed	MitoQ/salbutamol/intralipid, 20% intravenous emulsion	Insulin resistance
NCT05410873	Phase 2	106	Recruiting	MitoQ/placebo	Dilated cardiomyopathy
NCT00433108	Phase 2	30	Completed	MitoQ	Chronic hepatitis C
NCT05381454	Phase 1/Phase 2	80	Completed	MitoQ	Respiratory viral infection, antiviral treatment, COVID-19
NCT01167088	Phase 2	110	Terminated	MitoQ/placebo	Non-alcoholic fatty liver disease
NCT05373043	NA	300	Recruiting	MitoQ/placebo	Long-COVID
NCT05886816	Phase 2	112	Not yet recruiting	MitoQ/placebo	SARS-CoV infection, COVID-19
NCT02690064	NA	13	Active not recruiting	Acute antioxidant/chronic antioxidant/placebo	Cystic fibrosis

*Drp1* dynamin-related protein 1, *MitoQ* mitoquinone mesylate, *BQ-123* an endothelin receptor antagonist, *BH4* tetrahydrobiopterin, *L-NMMA* L-NG-monomethyl arginine, *COVID-19* corona virus disease 2019, *SARS-CoV* severe acute respiratory syndrome coronavirus, *NA* not applicable

### Dynasore

Dynasore, a non-competitive dynamin inhibitor, can inhibit Drp1 polymerization and obstruct Drp1 GTP hydrolysis [205], thereby suppressing mPTP channel opening and oxidative stress by maintaining calcium homeostasis [206]. Dynasore also reduces ROS accumulation by blocking transferrin receptor endocytosis in ischemia–reperfusion injury [207]. Furthermore, Dynasore can impede dynamin-mediated vesicle endocytosis and enhance the formation of mitochondrial antiviral signaling aggregates, thus blocking viral invasion and capsid formation [208]. Therefore, Dynasore has been used as an antiviral therapeutic strategy or vaccine adjuvant, exhibiting great clinical potential.

### Novel Drp1 inhibitors discovered by screening

Due to the rapid development of drug screening technologies, such as high-throughput and high-content screening approaches, some new Drp1 inhibitors are continuously being identified. Wu et al. [209] revealed 17 compounds with high predicted affinity to the GTPase domain of Drp1 through virtual screening of a chemical library and identified Drpitor1 and Drpitor1a as putative potent Drp1 inhibitors through in silico screening. Drpitor1 and Drpitor1a have higher potency than Mdivi-1 in inhibiting the GTPase activity of Drp1. Drpitor1a prevents mitochondrial fission and improves right ventricular diastolic dysfunction during cardiac ischemia- reperfusion injury [209]. Furthermore, Yang et al. [210] exploited high-content live-cell imaging to screen for mitochondrial fission inhibitors and developed a covalent compound termed mitochondrial division inhibitor (MIDI). MIDI does not affect Drp1 tetramerization nor Drp1 GTPase activity but does block Drp1 recruitment to mitochondria, which involves targeting the interaction of Drp1 with multiple receptors via covalent interaction with Drp1-C367 [210]. In addition, Rosdah et al. [211] identified DRP1i27 as the first small-molecule inhibitor that directly binds to human Drp1 (human isoform 3) via surface plasmon resonance and microscale thermophoresis approaches. Molecular docking suggested that DRP1i27 binds to the GTPase site of Drp1 with hydrogen bonds to residues Gln34 and Asp218. DRP1i27 was also reported to exhibit cytoprotective effects in human fibroblasts under ischemia–reperfusion injury [211].

## Conclusions and perspective

The findings of our and other research teams have confirmed that Drp1-mediated mitochondrial dysregulation occurs in multiple tissues and organs in various hypoxic-ischemic diseases. Oligomerization changes and modifications of Drp1 can influence various aspects of mitochondrial quality and cellular function during ischemia and hypoxia by binding with various proteins.

Based on the following key lines of evidence accumulated to date, we propose that mitochondrial quality regulation is a self-protection mechanism for adaptation to external stimuli. Both acute over-activation and chronic inhibition of Drp1-mediated mitochondrial regulation are detrimental to cellular function.

First, in a short period of mild ischemia and hypoxia or hypoxic preconditioning, Drp1-mediated mitochondrial fission is upregulated to provide adequate energy for maintaining normal cellular function, with the aim of slightly increasing the number of mitochondria and supplying tissues with more ATP [212, 213]. This process is accompanied by other compensatory mechanisms for mitochondrial quality regulation, such as decreased mitophagy and increased apoptosis, which function together to preserve healthy mitochondria in response to external stimuli.

Second, in acute severe hypoxic-ischemic injury, some mitochondria may experience a decompensatory response due to poor stress resistance, further aggravating tissue damage. Increased ATP production caused by constant upregulation of Drp1-mediated mitochondrial fission is accompanied by considerable ROS production. The short-term upregulation of ROS production that cannot be effectively cleared causes a chain reaction of mitochondrial dysfunction and mitochondrial calcium overload [214, 215], eventually consuming more ATP and leading to an overall decrease in ATP content, forming a vicious cycle.

Third, under prolonged ischemia and hypoxia, cells that remain viable gradually adapt to the hypoxic environment and exhibit a Drp1-mediated mitochondrial quality imbalance. This partially suppresses mitochondrial fission by altering Drp1 activity (e.g., enhanced Drp1-Ser637 phosphorylation), triggering a series of adaptive measures (e.g. increased mitophagy and reduced apoptosis), which allow sub-healthy mitochondria to survive longer by maintaining the basic energy supply. These adaptive measures facilitate tumor cell growth in the hypoxic microenvironment. At the cachectic stage of cancer, Drp1 expression and activity are either chronically low or suppressed [216].

Therefore, treatment strategies for acute hypoxic-ischemic injury, including shock, ischemia–reperfusion, and chronic ischemic-hypoxic injury associated with aging or a hypoxic tumor microenvironment, should not be limited to the unidirectional regulation of Drp1 but should instead address the dynamic maintenance of mitochondrial homeostasis. In the future, monitoring and targeting the Drp1-mediated mitochondrial quality imbalance would represent a novel therapeutic strategy against multiple organ damage in different hypoxic-ischemic diseases. Accordingly, we propose that drugs blocking Drp1 protein interactions are more suitable for clinical translation than catalytic inhibitors.

## Abbreviations

Akt: Protein kinase B
APC: Anaphase-promoting complex
ATP: Adenosine triphosphate
ACSL4: Acyl-coenzyme A synthetase long-chain family member 4
ADP: Adenosine diphosphate
AMPK: Adenosine monophosphate-activated protein kinase
AKAP121: A-kinase anchoring protein 121
BAX: Bcl-2 associated X
BCL2L13: BCL2-like 13
Bcl-xL: B-cell lymphoma-extra large
BSE: Bundle signaling element
CaMK: Calcium-calmodulin kinase
COVID-19: Corona virus disease 2019
COX: Cytochrome C oxidase
Cyto-Drp1: Cytoplasmic Drp1
CDK5: Cyclin-dependent kinase 5
Cdh1: Cadherin 1
DDP: Deafness dystonia peptide
Drp1: Dynamin-related protein 1
DRR: Dynamic-related region
DUSP6: Dual-specificity phosphatase 6
ER: Endoplasmic reticulum
ER-Mito contact: Contact between the endoplasmic reticulum and mitochondria
ER-Drp1: Drp1 distributed in the ER
ERK1/2: Extracellular-signal regulated kinase
FENDRR: FOXF1 adjacent non-coding developmental regulatory RNA
Fis1: Mitochondrial fission protein 1
FUNDC1: FUN14 domain-containing protein 1
GTP: Guanosine triphosphate
GDP: Guanosine diphosphate
GED: GTPase effector domain
GPX4: Glutathione peroxidase 4
GSK-3β: Glycogen synthase kinase-3β
HIF-1α: Hypoxia-inducible factor-1α
Hmox1: Heme oxygenase 1
HSP90: Heat shock protein 90
IMM: Inner mitochondrial membrane
INF2: Inverted formin 2
KIF5B: Kinesin family member 5B
KLC1: Kinesin light chain 1
LRRK2: Leucine-rich repeat kinase 2
Lyso-Drp1: Drp1 distributed in the lysosomes
MAPL: Mitochondrial-anchored protein ligase
MCU: Mitochondrial calcium uniporter
MD: Middle domain
MDVs: Mitochondrial-derived vesicles
Mdivi-1: Mitochondrial division inhibitor-1
MFF: Mitochondrial fission factor
MFN2: Mitofusin 2
MICU1: Mitochondrial calcium uptake 1
MiD: Mitochondrial dynamics protein
MIDI: Mitochondrial division inhibitor
MitoQ: Mitoquinone
Mito-Drp1: Mitochondrial Drp1
Miro1: Mitochondrial Rho GTPase 1
MLKL: Mixed lineage kinase domain-like protein
MMP: Mitochondrial membrane potential
MOD: Multiple organ dysfunction
MOM: Mitochondrial outer membrane
mNCX: Mitochondrial sodium-calcium exchanger
mPTP: Mitochondrial permeability transition pore
mRyR: Mitochondrial ryanodine receptor
mtDNA: Mitochondrial DNA
MUL1: Mitochondrial ubiquitin ligase 1
NLRP3: NOD-, LRR- and pyrin domain-containing protein 3
NOX: NADPH oxidase
NPC1: Niemann-Pick C1 protein
OCR: Oxygen consumption rate
OPA1: Optic atrophy 1
OTUD6A: Ovarian tumor-associated protease deubiquitinase 6A
p62: Sequestosome 1
PDH: Pyruvate dehydrogenase
PH: Pleckstrin homology
PI(4)P: Phosphatidylinositol 4-phosphate
PKA: Protein kinase A
PKI: PKA inhibitor
PINK1: PTEN-induced kinase 1
PGAM5: Phosphoglycerate mutase 5
PGC1: Proliferator-activated receptor gamma coactivator 1
ROS: Reactive oxygen species
RIP: Receptor interacting protein
ROCK1: RhoA/Rho-associated kinase 1
RaM: Rapid Mode of mitochondrial calcium uptake
SARS-CoV-2: Severe acute respiratory syndrome coronavirus 2
SENPs: SUMO-specific proteases
Shrm4: Shroom4
SIRT3: Sirtuin 3
Srv2: Suppressor of rho1 vacuole 2
TCA: Tricarboxylic acid
TBC1D15: TBC domain family member 15
TIMM8a: Translocase of inner mitochondrial membrane 8a
TOMM20: Translocase of outer mitochondrial membrane 20
UCP-2: Uncoupling protein-2
ULK1: Unc-51-like kinase 1
USP15: Ubiquitin-specific protease 15
VD: Variable domain
